# Molecular mechanism of HNF-1A–mediated *HNF4A* gene regulation and promoter-driven HNF4A-MODY diabetes

**DOI:** 10.1172/jci.insight.175278

**Published:** 2024-06-10

**Authors:** Laura Kind, Janne Molnes, Erling Tjora, Arne Raasakka, Matti Myllykoski, Kevin Colclough, Cécile Saint-Martin, Caroline Adelfalk, Petra Dusatkova, Stepanka Pruhova, Camilla Valtonen-André, Christine Bellanné-Chantelot, Thomas Arnesen, Petri Kursula, Pål Rasmus Njølstad

**Affiliations:** 1 Department of Biomedicine and; 2 Mohn Center for Diabetes Precision Medicine, Department of Clinical Science, University of Bergen, Bergen, Norway.; 3 Department of Medical Genetics and; 4 Department of Pediatrics and Adolescent Medicine, Haukeland University Hospital, Bergen, Norway.; 5 Exeter Genomics Laboratory, Royal Devon and Exeter NHS Foundation Trust, Exeter, United Kingdom.; 6 Department of Medical Genetics, Sorbonne Université, AP-HP, Pitié-Salpêtrière Hospital, DMU BioGeM, Paris, France.; 7 Monogenic Diabetes Study Group of the Société Francophone du Diabète, Paris, France.; 8 Clinical Genetics, Pathology and Molecular Diagnostics, University Hospital Skåne, Lund, Sweden.; 9 Department of Pediatrics, 2nd Faculty of Medicine, Charles University and University Hospital Motol, Prague, Czech Republic.; 10 Clinical Chemistry and Pharmacology, University Hospital Skåne, Lund, Sweden.; 11 Department of Surgery, Haukeland University Hospital, Bergen, Norway.; 12 Faculty of Biochemistry and Molecular Medicine & Biocenter Oulu, University of Oulu, Oulu, Finland.; 13 Section of Endocrinology and Metabolism, Children and Youth Clinic, Haukeland University Hospital, Bergen, Norway.

**Keywords:** Metabolism, Diabetes, Structural biology, Transcription

## Abstract

Monogenic diabetes is a gateway to precision medicine through molecular mechanistic insight. Hepatocyte nuclear factor 1A (HNF-1A) and HNF-4A are transcription factors that engage in crossregulatory gene transcription networks to maintain glucose-stimulated insulin secretion in pancreatic β cells. Variants in the *HNF1A* and *HNF4A* genes are associated with maturity-onset diabetes of the young (MODY). Here, we explored 4 variants in the P2-*HNF4A* promoter region: 3 in the HNF-1A binding site and 1 close to the site, which were identified in 63 individuals from 21 families of different MODY disease registries across Europe. Our goal was to study the disease causality for these variants and to investigate diabetes mechanisms on the molecular level. We solved a crystal structure of HNF-1A bound to the P2-*HNF4A* promoter and established a set of techniques to probe HNF-1A binding and transcriptional activity toward different promoter variants. We used isothermal titration calorimetry, biolayer interferometry, x-ray crystallography, and transactivation assays, which revealed changes in HNF-1A binding or transcriptional activities for all 4 P2-*HNF4A* variants. Our results suggest distinct disease mechanisms of the promoter variants, which can be correlated with clinical phenotype, such as age of diagnosis of diabetes, and be important tools for clinical utility in precision medicine.

## Abstract 2

Crystal structure and transcriptional activation studies identify how <em>HNF4A</em> P2 promoter sequence variants bind HNF-1A and highlight disease mechanisms of P2-driven HNF1A-&ndash;maturity-onset diabetes of the young.

## Graphical abstract

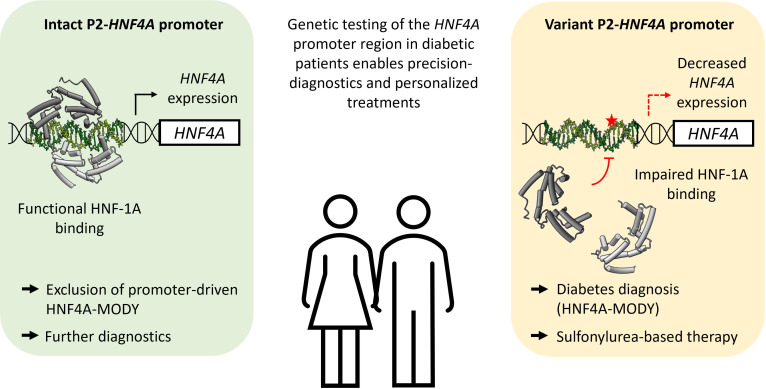

## Introduction

In order to improve diagnosis and treatment for patients with diabetes, the use of precision diabetes medicine has gained increased awareness (1). Knowledge of the exact molecular mechanism of disease is crucial for such practice. Monogenic diabetes types, such as neonatal diabetes and maturity-onset diabetes of the young (MODY), are single-gene disorders. Improved insight into the mechanism of disease has been important to enable precision diabetes treatment for several of these disorders, e.g., sulphonylurea agents for the treatment in K-ATP neonatal diabetes (2–4), HNF4A-MODY, and HNF1A-MODY (5–7). Using unsupervised learning methods, we have previously made an analytical framework for stratifying variants along the hepatocyte nuclear factor 1A (HNF-1A) protein phenotypic continuum to facilitate diagnostic interpretation (8). There are, however, still many unsolved puzzles, such as the molecular explanation for how variants in the promoter regions lead to disease and whether these are associated with phenotypic features.

**Figure 1:**
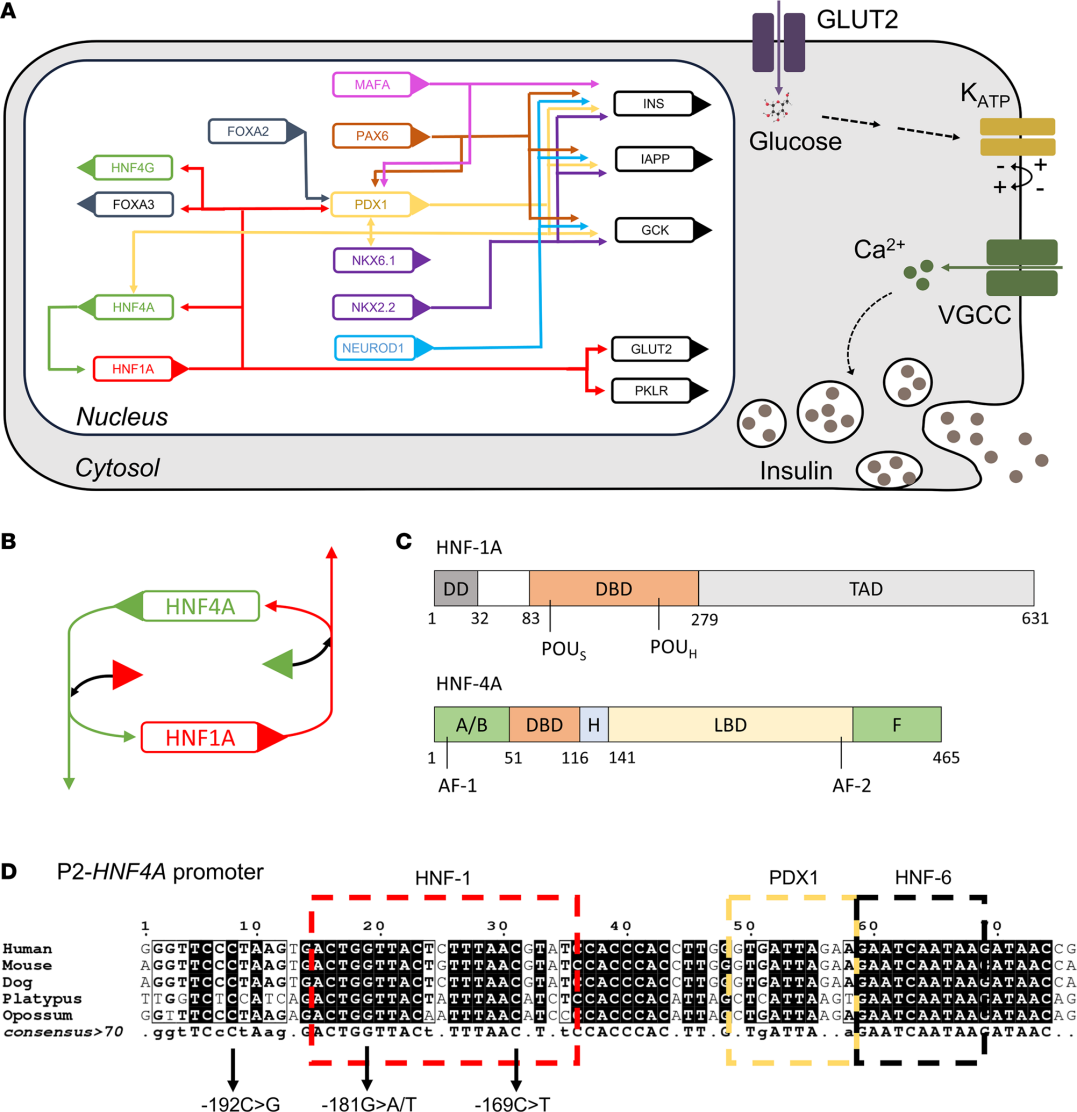
Transcriptional regulation in the pancreatic β cell. (**A**) Schematic overview of a pancreatic β cell. The endocrine cells perform glucose-stimulated insulin secretion, which consists of a signaling cascade including glucose uptake via the glucose transporter GLUT2, glucose metabolism and ATP production during glycolysis, ATP-induced blocking of K_ATP_ channels, depolarization of the cell membrane, opening of voltage-gated calcium channels (VGCC), and the calcium-induced exocytosis of insulin granules. Differentiated pancreatic β cells are controlled by a complex gene regulatory network, including the transcription factors HNF-1A, HNF-4A, FOXA2/3, pancreatic and duodenal homeobox 1 (PDX1), and paired box protein Pax-6 (PAX6), which together regulate target genes such as insulin (INS), glucokinase (GCK), and GLUT2. Sizes not to scale. (**B**) HNF-1A:HNF-4A regulatory circuit, in which each factor regulates the other by transcriptional control and protein-protein interactions. Boxes represent *HNF1A* and *HNF4A* genes, and triangles represent translated HNF-1A and HNF-4A proteins. Arrows indicate transcriptional control. (**C**) Domain overview of HNF-1A (top) and HNF-4A (bottom). DD, dimerization domain; DBD, DNA binding domain; POU_S_, POU-specific domain; POU_H_, POU homeodomain; TAD, transactivation domain; AF, activation function domain; H, hinge region; LBD, ligand binding domain; F, F domain; A/B, A/B domain (36, 69, 70). (**D**) Architecture and sequence conservation of the P2-*HNF4A* promoter, including transcription factor binding sites for HNF-1, PDX1, and HNF-6. Figure inspired by refs. 24, 32, and 52.

HNF-1A and HNF-4A form a crossregulatory loop, in which both factors regulate the respective other’s gene transcription (Figure 1, A and B) (9–12). Moreover, HNF-1A and HNF-4A interact physically and thereby further regulate gene transcription (Figure 1B) (13–15). The integrity of this HNF-1A:HNF-4A regulatory circuit is believed to be of exceptional importance, acting as a functional on-off switch for the genetic program of pancreatic β cells (16). Even though HNF-1A and HNF-4A are composed of different protein domains (Figure 1C) and exert specific gene regulatory functions (17), variants in the *HNF1A* and *HNF4A* genes lead to MODY forms of similar clinical phenotype (HNF1A-MODY and HNF4A-MODY, respectively) and are associated with neonatal hyperinsulinism (18–20). Both HNF1A-MODY and HNF4A-MODY are characterized by β cell dysfunction. That distinguishes them from type 1 diabetes caused by autoimmune destruction of the β cells and type 2 diabetes with defective insulin secretion by the β cells and the inability of insulin-sensitive tissues to respond appropriately to insulin. Importantly, patients with HNF1A/4A-MODY can be successfully treated with oral hypoglycemic agents, e.g., sulfonylureas, without the need for insulin injections (21, 22). The resemblance in phenotype of both MODY types supports a functional interaction between the 2 transcription factors.

HNF-4A belongs to the orphan nuclear receptor family (23). Besides its expression in pancreatic islets, HNF-4A is also expressed in kidney, liver, intestines, stomach, and skin (24), where it controls the transcription of genes involved in glucose, cholesterol, and lipid metabolism (23, 25). HNF-4A is considered a widely acting and constitutively active transcription factor as it was found to occupy promoters of 11% of hepatic genes and 12% of genes in pancreatic islets (17). The *HNF4A* gene is located on chromosome 20q and consists of 13 exons (23, 26, 27). Gene transcription is driven from 2 promoters (P1 and P2), and differential promoter usage and alternative splicing result in the temporal and tissue-specific generation of at least 12 isoforms (P1-derived HNF4α1–6 and P2-derived HNF4α7–12) (28). The usage of the P2 promoter was found to be pancreas specific, whereas P1-derived HNF-4A isoforms were mainly identified in hepatocytes (9, 23). The P2-*HNF4A* promoter presents an important link between MODY-associated genes, as it harbors transcription factor binding sites for HNF-1A, HNF-1B, PDX1, and HNF-6 (Figure 1D) (29). Pathogenic variants causing MODY have been reported in the P2 promoter and associated exon 1 sequence (30) but have not been identified to date in the P1 promoter.

Considering the importance of the HNF-1A:HNF-4A circuit and the intricate control of P2-derived HNF-4A isoforms in the pancreatic β cells, we set out to study the HNF-1A:P2 regulatory axis on a molecular level and to investigate the potential causality between P2 promoter variants and HNF4A-MODY development. We collected clinical data from different MODY registries across Europe and analyzed the phenotypes of patient groups harboring 2 of 4 P2-*HNF4A* variants in and close to the HNF-1A binding site: variants NM_175914.5 c.-169C>T (GRCh37 g.42984276C>T), c.-181G>A (GRCh37 g.42984264G>A), c.-181G>T (GRCh37 g.42984264G>T), and c.-192C>G (GRCh37 g.42984253C>G) (Figure 1D). In order to explain the obtained phenotypical data, we used approaches of biochemistry and molecular biology and compared the results to the clinical data. We solved a crystal structure of the HNF-1A DBD bound to an oligonucleotide with the P2 promoter sequence. We further established an in vitro platform to assess the binding of HNF-1A to different target promoters and used transactivation assays in cells to study the transcriptional activity of HNF-1A toward these targets. Finally, we used this system to compare the P2 wild-type (WT) promoter with the selected P2 variants, shedding light on disease mechanisms of P2-driven HNF4A-MODY diabetes.

## Results

### Clinical data on P2-HNF4A variant carriers.

We collected clinical data from individuals carrying variants in the P2-*HNF4A* promoter and the *HNF4A* coding region. These included 32 individuals from 13 families with a variant in the HNF-1A binding site (P2 -169C>T, P2 -181G>A, or P2 -181G>T), 31 individuals from 8 families that carried the P2 -192C>G variant, and 1,191 individuals with disease-causing variants in the *HNF4A* coding gene (Figure 1D, Supplemental Figure 5, Table 1, and Supplemental Table 1; supplemental material available online with this article; https://doi.org/10.1172/jci.insight.175278DS1). A total of 162 of the latter carried the c.340C>T (p.R114W) *HNF4A* variant, which is associated with a reduced penetrance (31).

**Figure 2:**
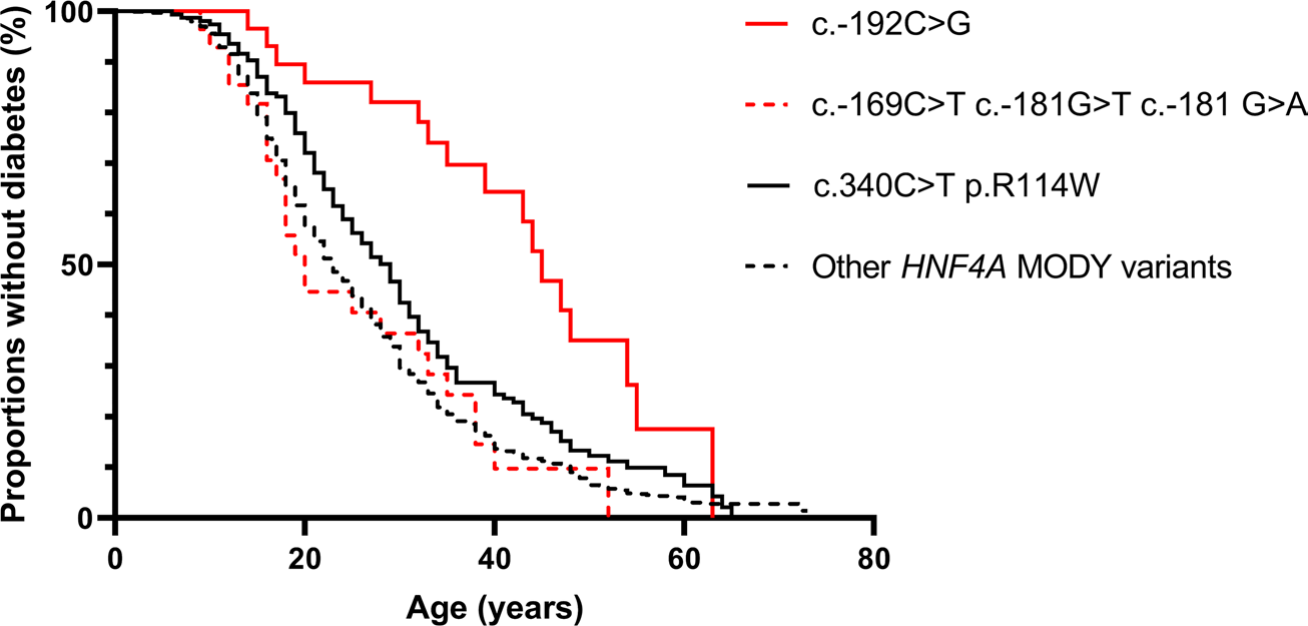
Kaplan-Meier plot demonstrating proportions of mutation carriers without diabetes at different ages for different *HNF4A* variants. Red lines represent P2-*HNF4A* promotor variants, while black lines represent coding variants in *HNF4A*. P2-*HNF4A* promotor variants within the HNF-1A binding site are merged into 1 group, as are all coding variants in *HNF4A* with the exception of the low-penetrance variant c.340C>T (31).

The c.-169C>T, c.-181G>A, and c.-192C>G P2-*HNF4A* promoter variants had previously been identified by genetic sequencing in patient cohorts and shown to cosegregate with MODY diabetes (9, 32, 33). While the nucleotide positions c.-169C and c.-181G are located within the HNF-1A binding region, the c.-192C nucleotide is located in the flanking region, 6 bp upstream of the HNF-1A binding site (Figure 1D). Comparing phenotypes for the 3 HNF-1A binding site variants versus the flanking region variant, age of diagnosis was 10.4 years higher for patients carrying the P2 -192C>G variant (Table 1). Hazard rate (HR) for age of onset of diabetes was significantly lower when comparing carriers of the c.-192C>G P2-*HNF4A* promoter variant with carriers of known disease-causing coding *HNF4A* variants (*P* < 0.001, Table 2 and Figure 2). This effect was also observed for carriers of the less penetrant c.340C>T (p.R114W) variant in the coding *HNF4A* gene, which had significantly lower HR (*P* < 0.001) compared with other *HNF4A* coding variants. In contrast, the merged group of carriers of P2-*HNF4A* promoter variants within then HNF-1A binding site (c.-169C>T, c.-181G>A/T) did not have significantly different HR when compared to carriers of coding *HNF4A* variants (Table 2 and Figure 2).

Female patients had significantly higher HR for development of diabetes when compared with males (*P* < 0.001), while HR for BMI was not significantly different from 1 (Table 2). There were 3 occurrences of neonatal hypoglycemia in the P2-*HNF4A* promotor variant carriers, each in a different mutation variant cohort (Table 1).

### HNF-1A binds to the P2-HNF4A promoter and activates P2-driven gene transcription.

We set out to characterize the identified P2-*HNF4A* (P2) promoter variants on a molecular level. Our initial aim was to establish a collection of techniques, which would allow us to quantitatively assess HNF-1A binding and gene transcriptional activity for the P2 promoter and given promoter variants. For reference, we included the promoter of the albumin gene, which is a well-established target of hepatic HNF-1A. The rat albumin (RA) promoter contains the palindromic consensus DNA sequence for optimal HNF-1A binding (GTTAATNATTAAC) (34) and has routinely been used to investigate HNF-1A DNA recognition and HNF-1A–mediated gene transcription (8, 35–37). Since the P2 promoter sequence deviates from this optimal consensus sequence in the RA promoter (Supplemental Figure 1A), we expected to observe differences in DNA recognition and transcriptional activity when comparing the 2 promoters.

**Figure 3:**
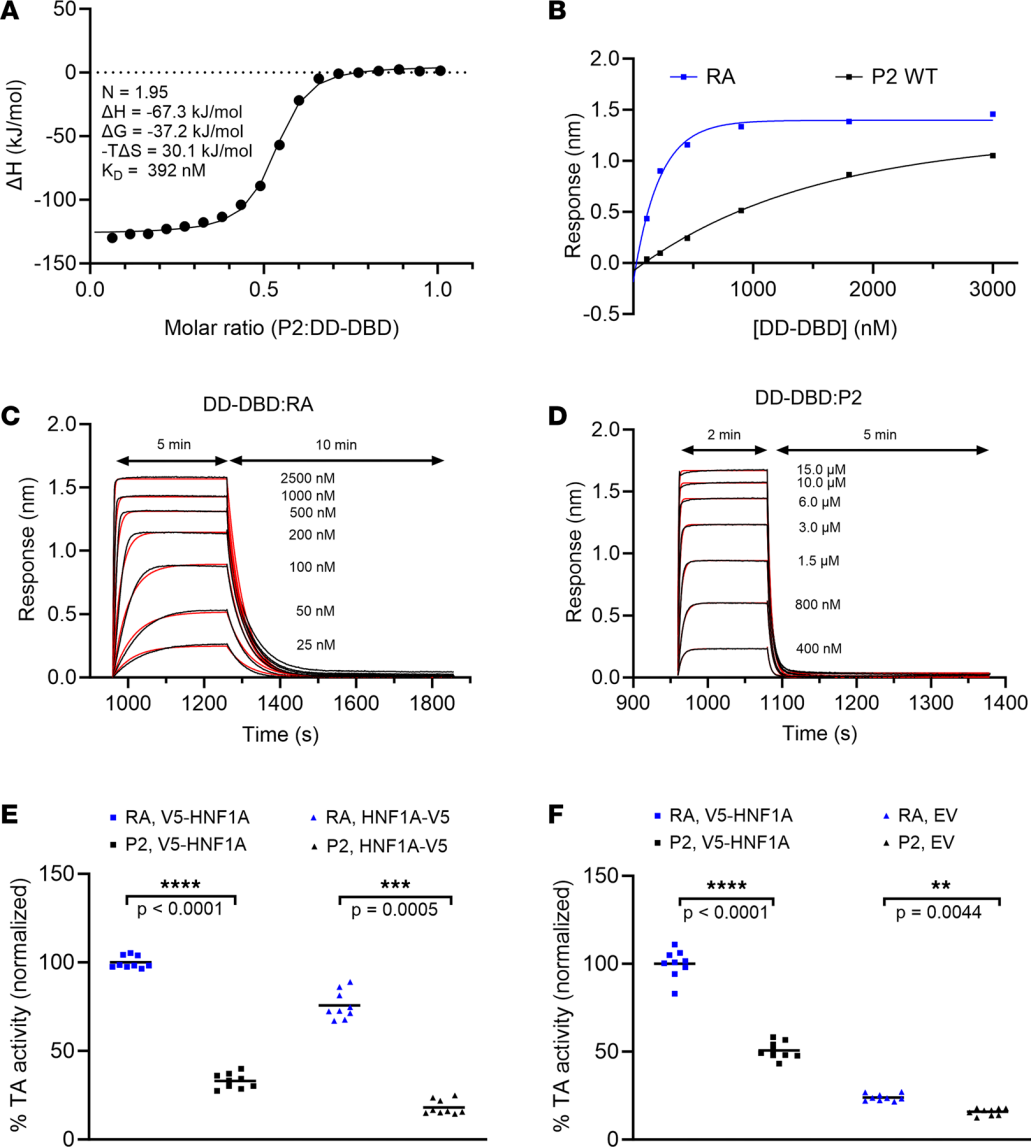
Biochemical and functional characterization of the HNF-1A:P2 interaction. (**A**) ITC titration of 50 μM DD-DBD with 250 μM P2 oligonucleotide. (**B**) Response curves from BLI measurements of DD-DBD:RA and DD-DBD:P2 interactions. Measurement series 1 is shown representatively. (**C** and **D**) Association and dissociation traces of DD-DBD:RA (**C**) and DD-DBD:P2 (**D**) binding. Raw traces and fits are shown in black and red, respectively. (**E** and **F**) Transactivation assay in HeLa (**E**) and MIN6 (**F**) cells. Normalized transcriptional activity of overexpressed V5-HNF-1A, HNF-1A-V5, or endogenous HNF-1A (when only an empty vector [EV] is transfected) toward the RA- or P2-controlled FL reporter gene. Individual data points are presented along with mean values. Significance levels used in nested 2-tailed *t* test analysis: **: *P* < 0.01; ***: *P* < 0.001; ****: *P* < 0.0001.

Indeed, when performing isothermal titration calorimetry (ITC) and biolayer interferometry (BLI) experiments, we observed clear differences between the RA and P2 promoters (Figure 3, A–D). ITC and BLI were performed using a purified protein harboring the DD and DBD of HNF-1A (HNF-1A 1-279, Figure 1C). We excluded the C-terminal TAD of HNF-1A in these assays, as this region is intrinsically disordered (38) and complicated the expression and purification of the target protein. The DD-DBD HNF-1A construct is dimeric in solution and exhibits high affinity toward a synthetic RA oligonucleotide (*K_D_* = 100 ± 50 nM), as previously demonstrated by ITC experiments (36). Identical measurements with a P2 oligonucleotide revealed a lower affinity (*K_D_* = 304 nM ± 134 nM), which was in accordance with our expectations because of the noncanonical HNF-1A binding site in the P2 promoter sequence (Figure 3A and Table 3). The ITC results also showed that DD-DBD bound the P2 double-stranded oligonucleotide in a 2:1 stoichiometric ratio (Figure 3A), indicating a similar binding mode as for the DD-DBD:RA interaction (36, 37).

We conducted BLI measurements to verify the observed result and to compare kinetic parameters of the DD-DBD:RA and DD-DBD:P2 interactions (Figure 3, B–D). BiotinTEG-labeled RA or P2 oligonucleotides were immobilized on a streptavidin-coated biosensor, and the interaction with DD-DBD was probed at different protein concentrations. An additional oligonucleotide with a randomized sequence was included as a negative control, for which no binding of DD-DBD was detected (Supplemental Figure 1C). The equilibrium responses of the RA and P2 association curves indicated that HNF-1A DD-DBD exhibited stronger binding to the RA oligonucleotide compared with the P2 oligonucleotide, as higher DD-DBD concentrations were required for the P2 sample to reach saturation in complex formation (Figure 3B and Supplemental Figure 1D). *K_D_* values for both complexes were extracted from the equilibrium response curves, which verified the lower affinity of HNF-1A toward the P2 oligonucleotide compared with the RA oligonucleotide (Supplemental Figure 1D and Table 3).

The association and dissociation traces were fit to a 1:1 binding model, assuming a 2-state binding reaction (Figure 3, C and D, and Supplemental Figure 1B). It is worth noting that the dissociation curves for the DD-DBD:RA interaction were best fit with a 1:2 heterogenous ligand model, indicating that intermediate dissociation steps might occur in the BLI measurement (Supplemental Figure 1B and Supplemental Table 2). Association and dissociation rate constants obtained from the 1:1 binding model (*k_on_* and *k_off_*, respectively) revealed a significant difference between the RA or P2 oligonucleotides, as DD-DBD association with RA was faster and dissociation from RA was slower compared with the association and dissociation steps for P2 (Figure 3, C and D; Table 3; and Supplemental Figure 1, E and F). The extracted rate constants were used to calculate *K_D_* values of the interactions, which again verified a higher affinity of HNF-1A toward the RA oligonucleotide compared with the P2 oligonucleotide (Table 3).

We further investigated the transcriptional activity of full-length HNF-1A toward the 2 promoters using transactivation assays in mammalian cells (Figure 3, E and F). Here, we used a luciferase-based reporter plasmid, in which a firefly luciferase (FL) gene was controlled either from the high-affinity RA promoter or from the P2 promoter, directly reporting on the transactivation potential of HNF-1A. We tested the transcriptional activity of N- and C-terminally V5-tagged HNF-1A toward these promoters in HeLa cells (Figure 3E). The transcriptional activity of HNF-1A was significantly higher for the RA-controlled FL gene compared with the P2-driven reporter, independent of the position of the V5-tag. The transcriptional activity toward the P2 promoter was approximately 40% of that toward the RA promoter (Figure 3E). A similar trend was observed in β-like MIN6 cells (Figure 3F) (39, 40). Here, the transcriptional activity of N-terminally V5-tagged HNF-1A was significantly reduced to about 50% with the P2-controlled FL gene, compared with the RA-controlled reporter gene. To test the transcriptional activity of endogenous HNF-1A in MIN6 cells, we performed the assay with a V5 expression plasmid devoid of HNF-1A (empty vector). Here, the luciferase signal was much lower compared with that of the samples with overexpressed HNF-1A, reflecting the relatively low expression levels of endogenous HNF-1A in MIN6 cells. Importantly, the reduction in transcriptional activity toward the P2 promoter was persistent, indicating that endogenously expressed HNF-1A is equally impaired in DNA binding as transiently overexpressed HNF-1A (Figure 3F).

In conclusion, HNF-1A binds the P2-*HNF4A* promoter with lower affinity than the RA promoter, which results in significantly reduced transcriptional activity in cultured cells. We demonstrate that biochemical analyses of HNF-1A DNA binding in vitro, alongside transactivation assays in cells, are a useful approach to assess the direct impact of single base pair changes within a given HNF-1A target sequence.

### Structural insights into promoter-specific DNA recognition by HNF-1A.

**Figure 4:**
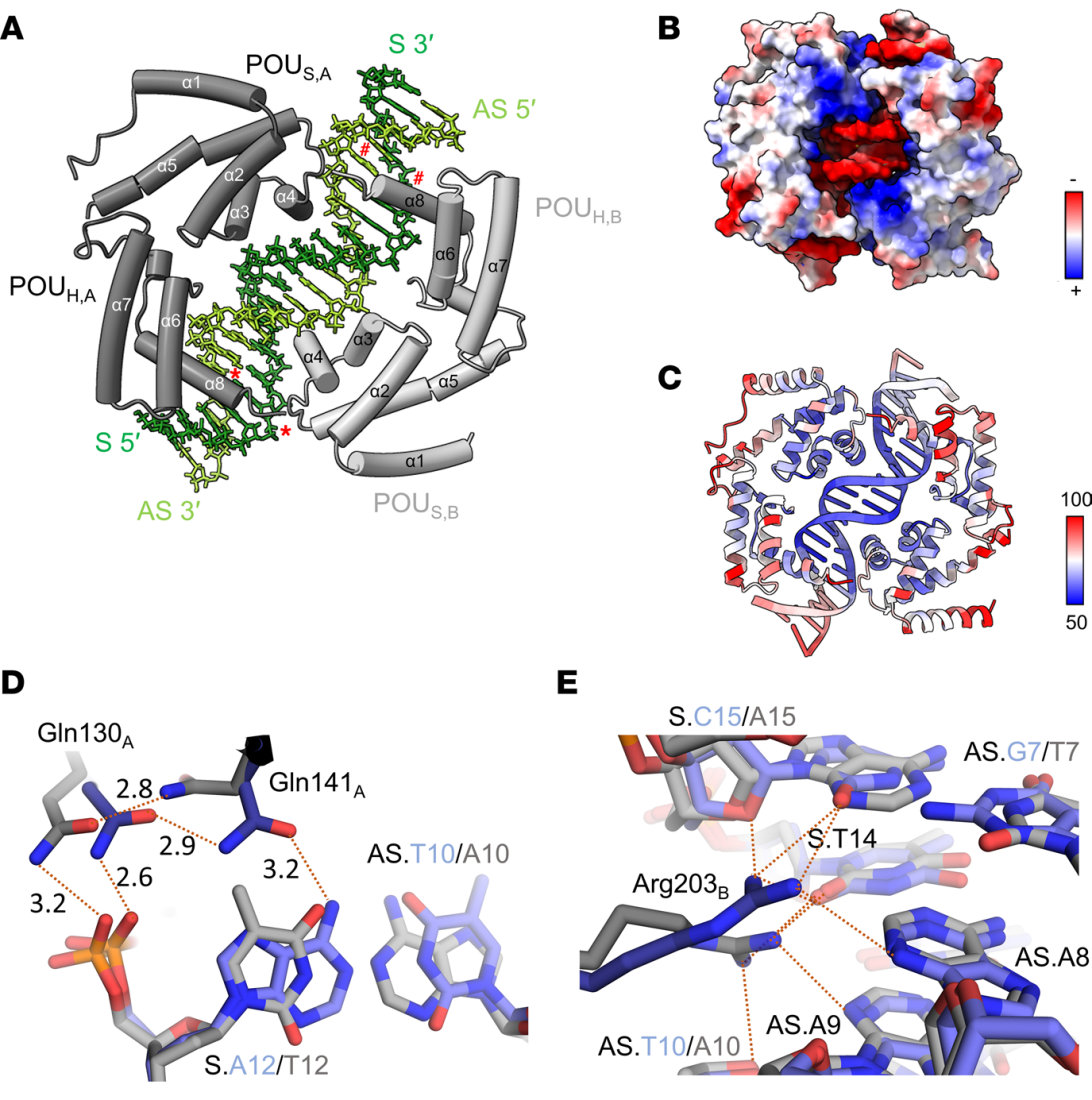
Structural analysis of the DBD:P2 complex. (**A**) Overall structure of a DBD dimer (gray) bound to a P2 oligonucleotide (green). Bases with red marking correspond to nucleotide positions of identified P2 promoter variants in patient cohorts (#: P2 -169C>T, *: P2 -181G>A/T). POU_S,A/B_, POU-specific domain of chain A/B; POU_H,A/B_, POU homeodomain of chain A/B; S, sense DNA strand; AS, antisense DNA strand. (**B**) DBD:P2 complex structure with surface coloring according to electrostatic potential. (**C**) DBD:P2 complex structure with coloring according to B-factor. (**D** and **E**) Detailed structural analysis of protein-DNA interactions, highlighting differences between the DBD:RA (blue) and DBD:P2 (gray) complex structures. Red dashed lines correspond to hydrogen bonds and ionic interactions, with the number representing the distance (in Å) between involved atoms.

Structural analysis of the HNF-1A DNA binding interface can shed light on specific molecular interactions and may explain the observed differences between the 2 promoters. We therefore recombinantly expressed and purified an HNF-1A construct harboring the DBD (HNF-1A 83-279, Figure 1C) and crystallized the DBD:P2 complex using a synthetic P2 oligonucleotide (Supplemental Table 3 and Figure 4). We refined the crystal structure to 2.3 Å resolution (Figure 4A). The overall architecture of the DBD:P2 complex was similar to the previously reported DBD:RA complex (Protein Data Bank [PDB]: 1IC8) (37). Two DBD molecules bind to 2 P2 DNA double helices from opposite sides along the helical axis (Figure 4A). Both the POU_S_ and the POU_H_ contribute to DNA recognition by HNF-1A. The residues at the protein-DNA interface are predominantly positively charged, allowing for electrostatic interactions with the negatively charged phosphate groups in the DNA backbone (Figure 4B). The majority of protein-DNA interactions are mediated by helices α3 and α4 in the POU_S_, as well as helix α8 in the POU_H_ (Figure 4A). Accordingly, amino acid residues and bases within these regions have comparatively low B-factors, correspondingly being in an ordered state, whereas amino acids and nucleotides at the edges of the complex exhibit higher B-factors, thus being more dynamic (Figure 4C).

A structural alignment of the DBD:RA and DBD:P2 crystal structures revealed a nearly identical conformation of DBD, with an average C_α_ atom root-mean-square deviation (RMSD) of 0.58 Å. A few differences in DNA recognition were observed (Figure 4, D and E). Helix α4, which is situated in the major groove of the DNA, and helix α3 are required for POU_S_-mediated DNA recognition (Figure 4A). In the RA-bound structure, the side chains of Gln130 in α3 and Gln141 in α4 of chain A form hydrogen bonds with adenine 12 of the RA sense strand (S.A12) via the phosphodiester backbone oxygen and the base, respectively (Figure 4D). The 2 residues form polar contacts with each other, which may stabilize the protein (Figure 4D). The S.A12>T12 nucleotide change in the P2 promoter sequence leads to a conformational change of the Gln141 side chain, likely due to changes in the base interactions. The polar interactions between the side chains of Gln130 and Gln141 are maintained, but the Gln141 side chain does not interact with the S.T12 base (Figure 4D). The electron density of Gln141 in DBD:P2 was less defined as in the DBD:RA structure, indicating increased dynamics due to the S.A12>T12 nucleotide change (Supplemental Figure 2). Other POU_S_-mediated DNA interactions include polar contacts between Arg131 (α3), His143 (α4), Asn149 (α4), and Lys158 (α5) side chains and the DNA backbone, which are also found in the RA-bound structure. In addition, Ser142 forms base-specific DNA contacts with adenine 8 of the antisense DNA strand (AS.A8) in both promoter types.

DNA recognition in the POU_H_ is mediated by helix α8, which is inserted into the major groove of the DNA (Figure 4A). Base-specific interactions are formed by the side chains of Asn266, Asn270, and Lys273, while DNA backbone interactions are mediated by Arg263. Arg203 and Lys205, located in the turn adjacent to helix α6, make additional contacts with the DNA. Arg203 of chain B is found in different conformations in the RA- and P2-bound structures (Figure 4E). In the RA-bound structure, Arg203 forms hydrogen bonds with the oxygen in the deoxyribose ring and the cytosine base of S.C15, the succeeding thymine base of S.T14, as well as the adenine base of AS.A8 in the antisense DNA strand. Arg203 in the P2-bound structure, however, adopts a differently oriented conformation, in which it appears to form a hydrogen bond with the thymine base of S.T14, as well as with AS.A9 and AS.A10 in the complementary strand (Figure 4E).

### Diabetes-associated P2-HNF4A variants exhibit reduced HNF-1A binding.

As our experimental pipeline had proven suitable for the comparison of the 2 HNF-1A target promoters, we turned to the biochemical analysis of the P2-*HNF4A* promoter variants of clinical interest. For in vitro assays with purified DD-DBD protein, we focused on the P2 variants harboring nucleotide changes within the HNF-1A binding site.

**Figure 5:**
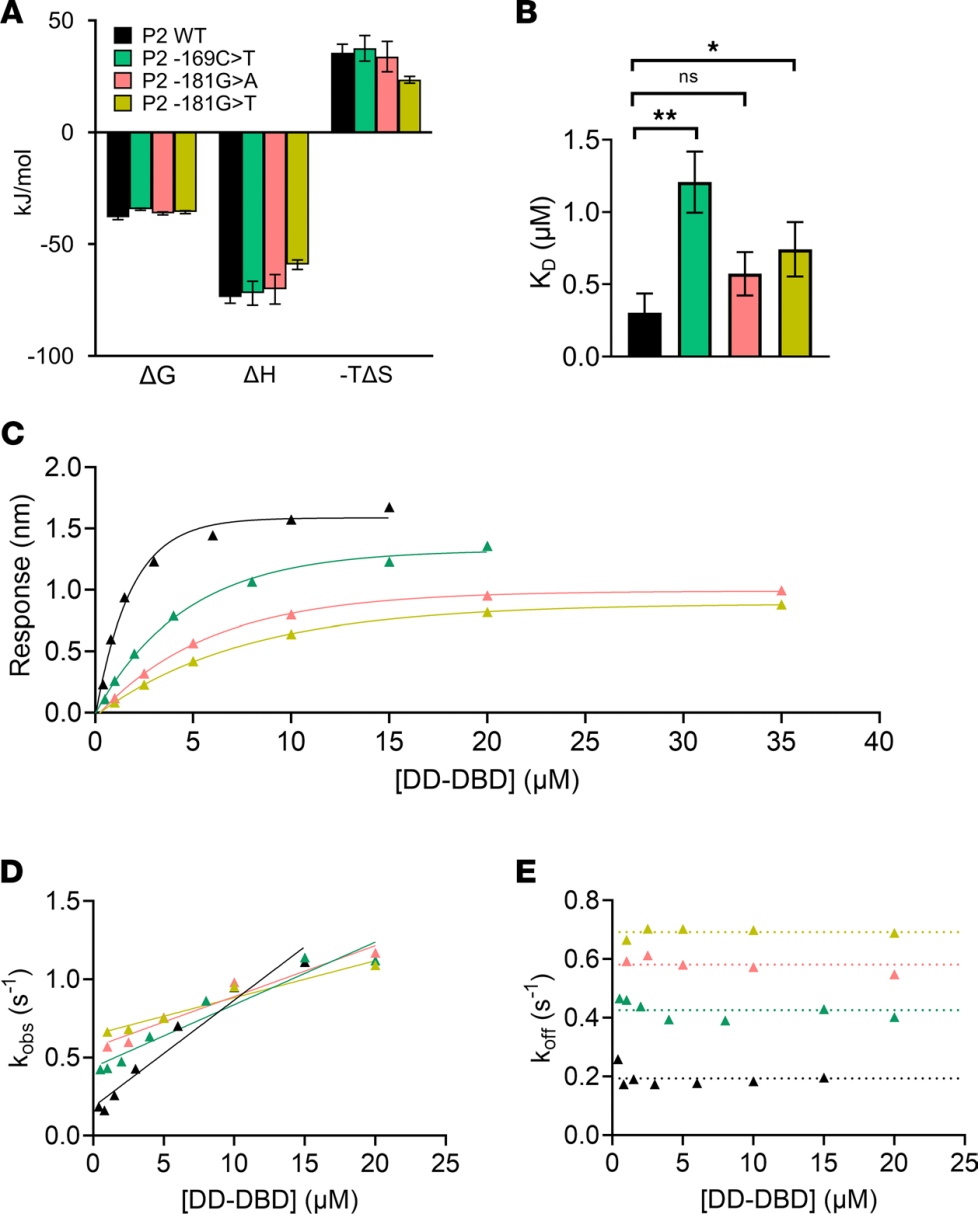
Thermodynamic and kinetic parameters of the DD-DBD:P2 WT and variant interactions. (**A**) Thermodynamic analysis of the DD-DBD:P2 WT and variant interactions by ITC measurements. Measurements were done in technical triplicates (*N* = 3). (**B**) *K_D_* values extracted from ITC measurements in **A**. (**C**) BLI response curves for P2 WT (black), P2 -169C>T (green), P2 -181G>A (pink), and P2 -181G>T (yellow). (**D**) Observed rate constants (*k_obs_*), extracted from association reaction traces in BLI measurements. Data were fitted to a linear function f(x) = *m* x + *n* (solid line), for which *m* corresponds to the association rate (*k_on_*) noted in Table 4. (**E**) Dissociation rates (*k_off_*), extracted from dissociation reaction traces in BLI measurements. The average value across [DD-DBD] is represented as dotted line and corresponds to *k_off_* noted in Table 4. (**C**–**E**) Measurement series 1 is shown representatively. Coloring according to panel **A**. Significance levels used in unpaired 2-tailed *t* test with Welch’s correction: *: *P* < 0.05; **: *P* < 0.01.

ITC revealed that the thermodynamic parameters of the binding reaction differed between P2 WT and the P2 variants (Figure 5A). We observed changes in both the enthalpic and entropic component, leading to an unfavorable change in the free binding energy of the P2 variant complexes (Figure 5A). All 3 tested variants led to an increased *K_D_* value, which was significant for the P2 -169C>T and -181G>T variants (Figure 5B and Table 4). The P2 -169C>T variant exhibited the strongest increase in *K_D_*, which was determined to 1.2 μM and was about 4 times as high as the *K_D_* of the DD-DBD:P2 WT interaction (*K_D_* = 0.3 μM). The *K_D_* values of the P2 -181G>A and P2 -181G>T interaction were intermediate, with 0.6 μM and 0.7 μM, respectively (Table 4).

BLI measurements verified the above observations (Figure 5, C–E, and Table 4). To ensure accurate measurements of *k_on_*, *k_off_*, and *K_D_* values, the DD-DBD concentration range needed to be increased for the P2 -169C>T and P2 -181G>A/T variants, as higher DD-DBD concentrations were required to reach the saturation of the binding response curves (Figure 5C). The *K_D_* values, extracted from the response curves, indicated that the binding of DD-DBD was weakened for the P2 variants compared with the P2 WT oligonucleotide (Table 4). The association and dissociation traces revealed that both the *k_on_* and *k_off_* rates of the interaction were affected by the point mutations (Figure 5, D and E, and Table 4). The strongest effects were observed for the P2 -181G>T variant, for which the *k_on_* rate was ~3 times lower and the *k_off_* rate ~3 times higher than for the P2 WT sequence. The resulting *K_D_* values from the kinetic analyses reflect the impaired HNF-1A binding, as the *K_D_* values were 5 (P2 -169C>T) to 10 (P2 -181G>T) times higher than for the P2 WT sample (Table 4).

Our in vitro studies demonstrate that the P2 -169C>T and P2 -181G>A/T point mutations had a negative effect on HNF-1A binding, which in turn may lead to an impaired gene activation by HNF-1A in the pancreatic β cells.

### P2 nucleotide changes modulate protein-DNA interactions in the DBD:P2 complex.

Next, we set out to investigate whether the P2 variant-induced changes in HNF-1A DNA binding kinetics and strength were accompanied by structural changes in the protein-DNA interface. We therefore crystallized the protein-DNA complexes and determined their structures at 3.2 Å (P2 -169C>T) and 2.8 Å (P2 -181G>A, P2 -181G>T) (Supplemental Table 3).

**Figure 6:**
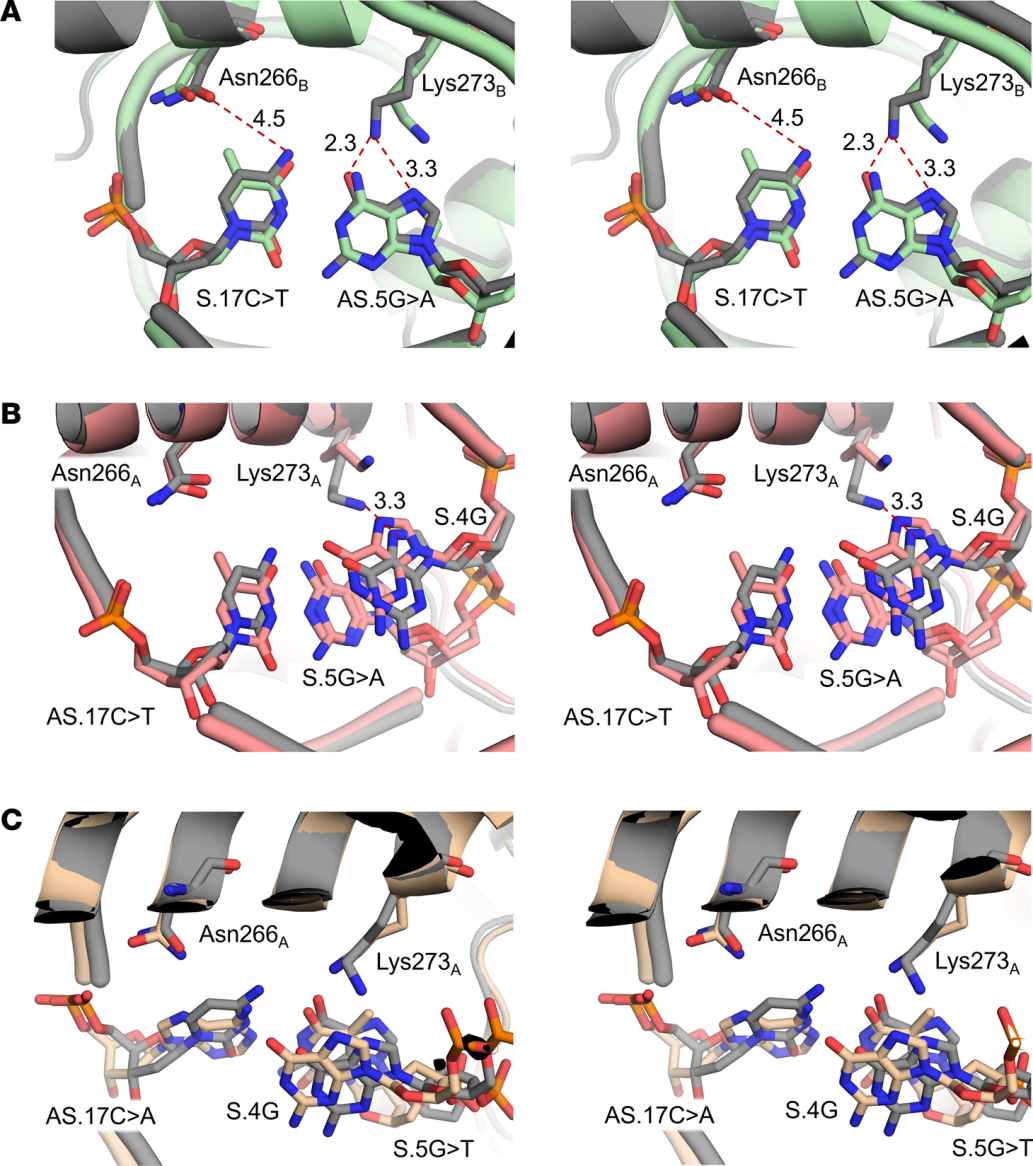
Structure analysis of DBD:P2 variant complexes. (**A**) Superposition of DBD:P2 WT (gray) and DBD:P2 -169C>T (green) complex structures. Magnified view into residue base interactions of Asn266 and Lys273 in helix α8 chain B. (**B** and **C**) Superposition of DBD:P2 WT (gray) and DBD:P2 -181G>A (pink, **B**) or DBD:P2 -181G>T (yellow, **C**) complex structures. Magnified view into residue base interactions of Asn266 and Lys273 in helix α8 of chain A. Structures are shown as stereo-view images.

A structural alignment of the 3 mutant complex structures and the DBD:P2 WT structure showed that there was no substantial change in overall conformation of the protein or the oligonucleotide. The average RMSD of the DBD C_α_ atoms of the superimposed P2 WT and P2 variant structures was 0.48–0.84 Å. When studying protein-DNA interactions close to the respective site of nucleotide mutation, we observed minor rearrangements of the involved recognition residues (Figure 6, A–C). Interestingly, the mutation sites in all 3 P2 variants were in proximity to the same residues, which were Asn266 and Lys273 in helix α8. As the P2 promoter sequence is nearly palindromic and the P2 -169C>T and P2 -181G>A variants affect nucleotides at opposite ends of the oligonucleotide (Figure 1D and Table 5), the Asn266 and Lys273 residues were located in either of the 2 DBD chains (Figure 4A).

In the P2 WT structure, both Asn266 and Lys273 of chain B form hydrogen bonds with cytosine and guanine bases, respectively (Figure 6A). The base changes occurring in the P2 -169C>T variant likely disrupt the favorable hydrogen bond network with the bases, e.g., by introducing a hydrophobic methyl group upon S.17C>T mutation, which may weaken the interaction between Asn266_B_ and the base (Figure 6A). This is reflected by an increased flexibility of this residue, which is apparent from a less defined electron density map of Asn266_B_ in the P2 -169C>T complex structure compared with the P2 WT complex structure (Supplemental Figure 3). Lys273_B_ in the P2 WT complex structure forms a hydrogen bond with the guanine base in the complementary strand, which is likely weakened by the AS.5G>A mutation (Figure 6A). The electron density of the Lys273_B_ side chain was only weakly defined (P2 WT complex structure) or was absent (P2 -169C>T complex structure), indicating a high conformational flexibility of this residue (Supplemental Figure 3).

The mutation of P2 -181G at the opposite site of the oligonucleotide had a similar effect (Figure 6, B and C) in the DBD chain A. While Asn266_A_ and Lys273_A_ did not undergo any major conformational changes in the P2 -181G>A variant, the AS.17C>T base change likely leads to unfavorable interactions upon introduction of a hydrophobic methyl group (Figure 6B). Lys273_A_ in the P2 WT structure forms a hydrogen bond with the guanine base S.4G (Figure 6B). However, the weakly defined electron density in both structures suggests flexibility of this residue (Supplemental Figure 4). Similar observations were made in the P2 -181G>T variant structure (Figure 6C and Supplemental Figure 4). The substitution of a guanine with a thymine (S.5G>T) leads to an introduction of a hydrophobic methyl group, likely leading to unfavorable interactions with Lys273_A_ (Figure 6C).

### Computational predictions of P2-HNF4A variant-induced effects.

We utilized computational predictions to test whether a bioinformatics approach could recapitulate our observed results and indicate whether the single nucleotide changes in the P2 promoter would disturb or improve the canonical HNF-1A binding site. We used the FABIAN-variant prediction algorithm, which is designed to identify transcription factor binding sites in a given nucleotide sequence and to predict to which extent a DNA sequence variant affects binding of these transcription factors. The algorithm utilizes position weight matrices and transcription factor flexible models to accomplish this task. These metrics are derived from experimentally verified binding sites (41).

The FABIAN-variant algorithm identified an HNF-1A binding site in the P2 WT sequence. Numerous other transcription factors were identified to bind to the promoter sequence, such as HNF-1B (42) and HMBOX1 (43) with POU domains similar to those of HNF-1A. When comparing the P2 WT sequence with the P2 variants of interest, the algorithm predicted changes in the binding capacity of HNF-1A (Table 6). The 3 variants within the DBD binding site (P2 -169C>T, P2 -181G>A, P2 -181G>T) were predicted to have a minor negative effect on HNF-1A binding to the DBD binding site (bp P2 -165 – P2 -185). Interestingly, the results were more profound when analyzing the variants in the context of the upstream nucleotides (P2 -165 – P2 -199), for which the P2 -181G>A and P2 -181G>T variants were predicted to impair HNF-1A binding to a greater extent than the P2 -169C>T variant (Table 6). These results demonstrate that the upstream nucleotides outside of the DBD binding site may contribute to HNF-1A binding. Finally, the FABIAN-variant prediction suggested an increase in HNF-1A binding ability upon introduction of the P2 -192C>G variant.

### P2-HNF4A variants cause a change in HNF-1A transcriptional activity in cells.

**Figure 7:**
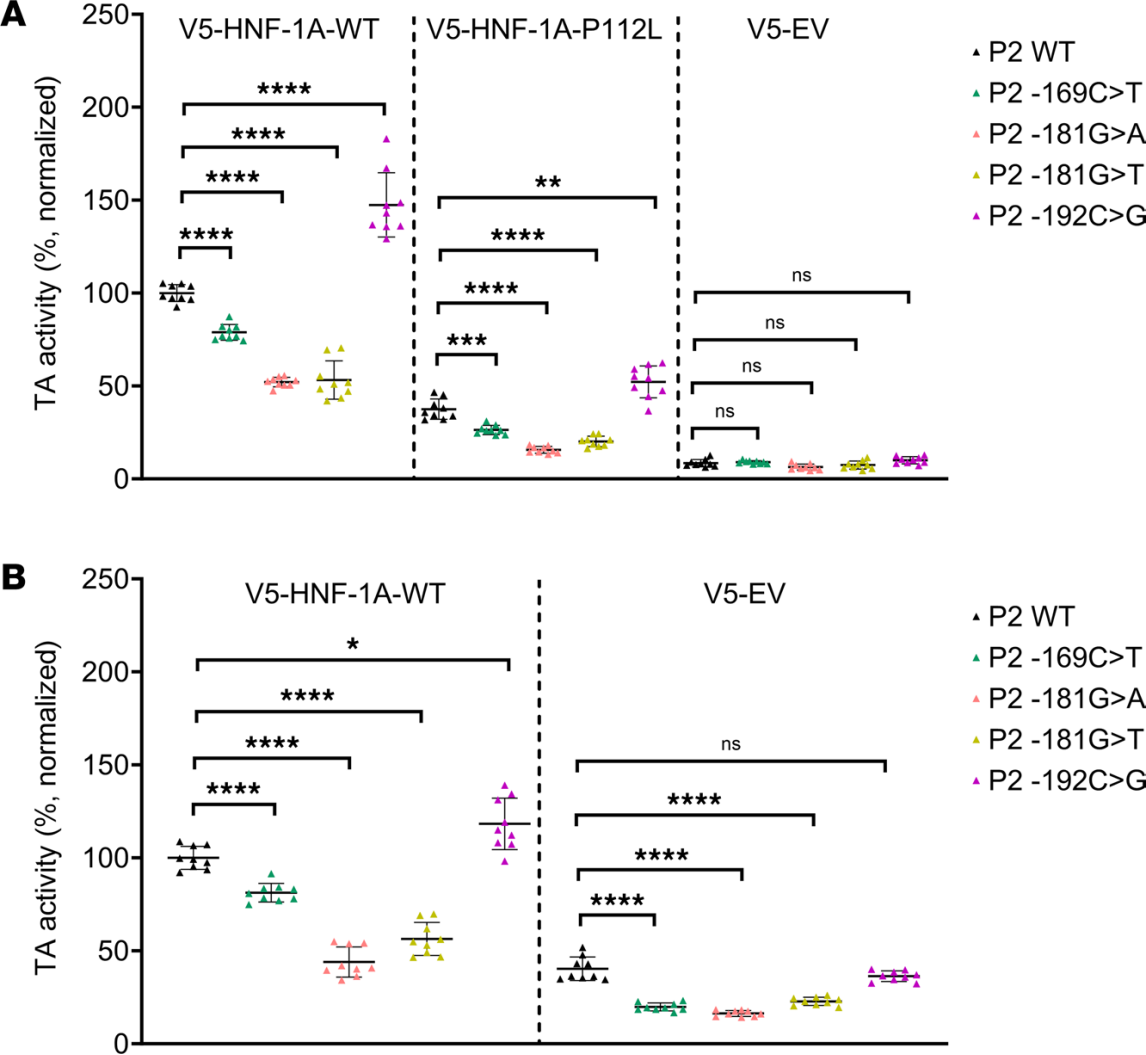
Transactivation assays probing for HNF-1A transcriptional activity toward the P2-*HNF4A* promoter variants. HeLa (**A**) and MIN6 (**B**) cells. Three HNF-1A expression plasmids were included (V5-HNF-1A-WT, V5-HNF-1A-P112L, V5-EV) to probe transcriptional activities of HNF-1A toward reporter genes controlled by P2 WT (black), P2 -169C>T (green), P2 -181G>A (pink), P2 -181G>T (yellow), and P2 -192C>G (magenta). All values are normalized to SV40-RL internal control activities and to V5-HNF-1A-WT activities with the P2 WT reporter. All measurements were performed as biological triplicates with 3 technical replicates. Individual data points are presented along with mean values. Significance levels used in 1-way ANOVA (Dunnett T3) analysis: *: *P* < 0.05; **: *P* < 0.01; ***: *P* < 0.001; ****: *P* < 0.0001.

Finally, we aimed to investigate the impact of the P2-*HNF4A* promoter variants on the level of P2-driven gene transcription. We used the dual-luciferase reporter assay described above, now measuring the transcriptional activity of full-length HNF-1A toward the selected P2 promoter variants (Figure 7). Here, we also included the P2 -192C>G variant, which is located upstream of the HNF-1A binding site (Figure 1D) and may be causing diabetes via a distinct molecular mechanism. In contrast to in vitro experiments with purified DBD or DD-DBD, this assay allowed us to study DNA binding in the context of the full-length HNF-1A protein, as the expression constructs also included the intrinsically disordered TAD of HNF-1A (38). Due to the position of the P2 -192C>G mutation, we speculated that the TAD of HNF-1A may play additional roles in modulating gene transcription and may be affected in functionality by the P2 -192C>G mutation.

We performed reporter assays in HeLa and MIN6 cells (Figure 7). To rule out any P2 variant-specific effects on endogenous transcription factors, we included control samples, in which HeLa cells were cotransfected with an empty control vector. These samples did not show any significant differences between the P2 promoter reporters, verifying that the experimental approach was sensitive to HNF-1A–specific effects (Figure 7A). We tested the transcriptional activity of WT HNF-1A and that of the HNF-1A P112L variant, which is a pathogenic HNF1A-MODY variant that is frequently used as positive control in HNF-1A gene reporter assays (8, 36, 44, 45). As expected, the overall transcriptional activities of HNF-1A P112L were lower than HNF-1A WT activities. For both expression plasmids, we observed a statistically significant decrease in normalized HNF-1A transcriptional activity for the 3 P2 variants within the HNF-1A binding site (P2 -169C>T, P2 -181G>A, P2 -181G>T) compared with the P2 WT reporter, which was in accordance with our in vitro data (Figure 7A). Surprisingly, the samples transfected with the P2 -192C>G variant reporter produced increased levels of FL protein compared with the P2 WT reporter samples (Figure 7A).

To probe P2 variant-induced changes of HNF-1A transcriptional activity in a β cell–like environment, we performed the same assay in MIN6 cells (Figure 7B). Here, we tested transcriptional activities of overexpressed and endogenous HNF-1A WT protein. The results were in accordance with the HeLa cell system. The transcriptional activity of HNF-1A toward the P2 -169C>T, P2 -181G>A, and P2 -181G>T variants was significantly reduced compared with the P2 WT-driven reporter genes, and this transcriptional impairment occurred for both overexpressed HNF-1A and endogenous HNF-1A in MIN6 cells. We observed a weak trend of P2 -192C>G producing increased FL levels compared with the P2 WT promoter for overexpressed HNF-1A, but the difference was absent when probing for activities of endogenous HNF-1A (Figure 7B).

## Discussion

With the aim to study potential diabetes-causing P2-*HNF4A* promoter variants on the molecular level, we established a set of techniques, which allowed us to probe HNF-1A binding in vitro and HNF-1A transcriptional activity in cells. While ITC and BLI were complementary methods to assess protein-DNA binding affinities, we were also able to extract kinetic parameters of the interactions. We observed impaired HNF-1A binding for 3 P2 variants of interest (Figure 5), which was verified by the transactivation assays in mammalian cells (Figure 7). BLI and ITC are generally underrepresented in studies on disease-causing DNA variants, and genetic approaches at the population level and molecular biological methods are the predominant choice to investigate disease causalities (46). Our study demonstrates that in vitro measurements can provide additional knowledge on direct interactions between transcription factors and their target DNA sequences and aid in the accurate classification of variants in promoter regions of disease-associated genes.

The ITC and BLI data were in agreement when comparing the P2 WT oligonucleotide with the 3 investigated promoter variants. However, the absolute *K_D_* values did differ somewhat between the 2 methods (Tables 3 and 4). The major difference between the 2 methods is that the DNA oligonucleotide is immobilized on a surface in BLI, while in ITC the molecules are free in solution. An additional explanation for the discrepancies between the BLI and ITC results could be the use of a buffer containing the blocking agents bovine serum albumin (BSA) and Tween 20, which was required to prevent unspecific binding to the biosensor surface. Our results highlight the need for a relative comparison between different oligonucleotide variants using the same method, as well as orthogonal approaches to verify the obtained results.

Our experimental approach could be improved by expanding the in vitro assays to use full-length HNF-1A and oligonucleotides harboring the flanking regions of the DBD binding site of the promoter. It has been shown that disordered regions of multidomain transcription factors are involved in DNA binding and modulate DNA binding specificity (47, 48) and that the flanking regions of the binding motif influence the search dynamics of transcription factors (49). This improvement would allow us to include the P2 -192C>G variant in the ITC and BLI assays and permit a direct comparison with the transactivation assays. To this end, however, we were not able to express and purify the full-length HNF-1A protein at sufficient yield and quality.

We solved the crystal structure of the DBD:P2 complex and 3 variants thereof. Structural analysis of the DBD:P2 variant complexes revealed that both c.-169C and c.-181G bases were recognized by the same residues, Asn266 and Lys273 in helix α8. Even though we observed only minor variant-induced changes in conformation of the protein residues or nucleotides, the structures help explain the decrease in binding affinities observed in ITC and BLI (Figure 6). It was surprising that the identified P2 variants were limited to this area of the P2:DBD interface (Figure 4A). Additional mutation sites within the HNF-1A binding site may exist, but these might not cause a defect in HNF-1A binding and β cell function and thus will not be reported by diabetes diagnostics laboratories (50).

The kinetic analysis on the DD-DBD:P2 variant interaction revealed that the variants within the DBD binding site exhibited decreased association and increased dissociation constants compared with the P2 WT oligonucleotide (Figure 5). This observation is biologically relevant, as it reflects on how long HNF-1A may reside at the promoter during genome scanning and is related to the potential of HNF-1A to recruit coactivating factors, such as CBP/p300 and P/CAF (51). Taken together with the affinity measurements and transactivation activities of HNF-1A, our data strongly suggest that an impairment of HNF-1A–mediated gene transcription is causative for MODY in these patient groups. A disturbance in the HNF-1A:HNF-4A regulatory loop may lead to a dramatic change in expression levels of both factors and subsequently propagate through the remaining gene regulatory network (Figure 1A). The P2 -169C>T and P2 -181G>A variants have previously been reported to cosegregate with diabetes, but the molecular mechanisms were not fully established (9, 32). Wirsing et al. showed that HNF-1B, a transcription factor paralog of HNF-1A (42), was impaired in transcriptional activity in a P2 reporter assay when the P2 -169C>T mutation was introduced (32). Importantly, HNF-1A and HNF-1B are expressed at different developmental stages, with HNF-1B being detected in the developing pancreas and HNF-1A being expressed and essential for the mature pancreatic β cell in the adult pancreas (52). Considering these expression patterns and the age of onset for MODY, we believe that an impaired HNF-1A–mediated gene transcription is likely the disease-causing factor, even though an involvement of HNF-1B in disease predisposition or progression is possible.

To our surprise, the P2 -192C>G variant stood out from the investigated P2 variants because of its enhancing effect on HNF-1A transcriptional activity in HeLa cells (Figure 7A). These data were in accordance with computational predictions, indicating that the flanking region of the HNF-1A binding site may contribute to DNA binding (Table 6). As HNF-1A and HNF-4A form a crossregulatory loop and together regulate an extensive transcriptional network in the pancreatic β cells (Figure 1A), an upregulation of *HNF4A* expression may be disrupting the precisely tuned balance of transcription factor levels and could cause β cell dysfunction. A recent study on the HASTER promoter of long noncoding RNAs transcribed from the antisense strand of the *HNF1A* gene supports this hypothesis (53). A positive regulation of P2-*HNF4A* expression by the P2 -192C>G variant may lead to similar effects, where a disturbed HNF-4A:HNF-1A regulatory loop may cause major changes in target gene expressions of both factors. Alternatively, the variant may act via a different route, affecting the binding and gene transcriptional activity of another, yet undefined, transcription factor. Such an effect would not have been uncovered in our studies, as we performed the transactivation assays exclusively with HNF-1A expression plasmids.

The P2 -192C>G dependent increase of HNF-1A transcriptional activity was mainly observed in HeLa cells (Figure 7), indicating that MIN6 cells may possess an additional regulatory mechanism, which tones down the variant-induced potentiation of transcriptional activity. An example of such a regulatory mechanism could be a β cell–specific interaction partner of HNF-1A. As proposed by Ferrer et al., a misregulation of the β cell transcriptional network could drive the cells into a nonfunctional state, and the average age of disease onset could be a function of the probability to reach this state (16). Our clinical and experimental data allow us to speculate that regulatory processes in the young adult mask the potentiating effect of the P2 -192C>G variant but that this protective effect may disappear during aging and thus explain a later disease onset for this variant. If age-related genetic effects play a role, this hypothesis implicates that the P2 -192C>G variant is not a typical MODY variant, but rather a variant with reduced expression of the phenotype. The phenotype of variant carriers (Tables 1 and 2) and the segregation pattern (Supplemental Figure 5, Family 14) suggest that the variant leads to HNF4A-MODY with reduced penetrance in addition to a delayed onset of disease and not being a risk factor for type 2 diabetes development. We demonstrate higher median age at onset of diabetes and significantly lower HR for early diabetes onset in the P2 -192C>G variant carriers, supporting our results being an important tool for precision medicine related to genetic counseling and prediction. It should be noted that the statistics used do not account for family relations between patients. An additional limitation of our clinical analysis is that different laboratories, with their own data acquisition practices, contributed to this multinational study.

In conclusion, we demonstrate that in vitro studies provide useful information during the classification process of variants in promoter regions of genes. This information can be used in precision medicine related to diagnostics and prediction. We encourage the inclusion of such regulatory regions in the sequencing routines of diagnostic laboratories, enabling an improved diagnosis of MODY and the acquisition of extensive genetic data for such genome regions. Knowledge about transcriptional networks and subcircuits is important to understand β cell function, which will enable future research efforts to intervene in disease progression and to generate functional β cells de novo.

## Methods

### Sex as a biological variable.

Variant carriers in our study are both females and males, and differences between sexes are reported.

### Clinical data.

We searched the Norwegian MODY Registry for individuals carrying a heterozygous sequence variant in the P2-*HNF4A* promoter. Subsequently, we approached the Monogenic Diabetes Variant Curation Expert Panel (https://clinicalgenome.org/affiliation/50016/) and asked whether any of the members had access to and approval to share information detailed below of individuals carrying any of the 4 described P2-*HNF4A* variants. Panel members working at laboratories in Exeter (United Kingdom), Paris (France), Lund (Sweden), and Prague (Czech Republic) provided relevant clinical information. For reference, the centers also provided information on patients with disease-causing variants in the coding part of *HNF4A*. Clinical and biological characteristics and family history were provided by clinicians at time of referral. We collected information in relation to other family members of each personʼs pedigree, age at investigation, sex, age at diagnosis, BMI, glycemic status (diabetes, impaired fasting glucose, or normoglycemic), HbA1c, treatment, and episodes of neonatal hypoglycemia (Table 1 and Supplemental Figure 5). The suspicion of a MODY diagnosis was made by the referring clinician.

### Protein constructs, expression, and purification.

Two HNF-1A protein constructs, omitting the TAD, were used in this study (36). HNF-1A 1-279 contained the DD and the DBD, while construct HNF-1A 83-279 contained only the DBD.

Proteins were expressed in *E*. *coli* Rosetta (DE3) at 20°C for 20 hours with 1 mM isopropyl β-d-1-thiogalactopyranoside, using Luria-Bertani growth medium. Bacteria were resuspended in 50 mM HEPES (pH 7.5), 500 mM NaCl, 1 mM DL-dithiothreitol (DTT), 20 mM imidazole, 0.1 mg/mL lysozyme from chicken egg white (Merck), 1× cOmplete EDTA-free protease inhibitor cocktail (Roche), and 1 mM phenylmethylsulfonyl fluoride. Bacterial cell lysis was performed by ultrasonication (7 minutes, 25 W, 1-second on/off cycles), and cell lysates were cleared by centrifugation (16,000*g*, 4°C, 10 minutes). Initial purification of His_6_-tagged proteins was achieved by metal ion affinity chromatography (IMAC) using a Ni-NTA column. Eluted proteins were dialyzed overnight (20 mM HEPES pH 7.5 [DD-DBD]/20 mM Tris-HCl pH 8.5 [DBD], 500 mM NaCl, 1 mM DTT), during which Tobacco-Etch Virus protease was added for proteolytic removal of the His_6_-tag. Cleaved protein was separated from uncleaved protein in a second IMAC step using a gradient of low imidazole concentrations. Fractions containing cleaved protein were pooled, concentrated, and gel-filtrated using a Superdex 75 pg 16/60 (GE Healthcare, now Cytiva) column in 20 mM HEPES pH 7.5, 500 mM NaCl, and 1 mM tris(2-carboxyethyl)phosphine (TCEP). Size-exclusion chromatography elution fractions were concentrated, snap-frozen in liquid N_2_, and stored at −80°C.

### Oligonucleotides used in in vitro binding assays.

Oligonucleotides, corresponding to the sequences of the HNF-1A binding site in the RA promoter (37), P2-*HNF4A* promoter (P2) (32), or corresponding variants thereof, were purchased from TAG Copenhagen (Table 5). Complementary forward and reverse oligonucleotides were dissolved in 10 mM Tris-HCl pH 8.0, 50 mM NaCl, and 1 mM EDTA and mixed in an equimolar ratio. Oligonucleotides were annealed to double-stranded DNA (dsDNA) by incubation at 95°C for 5 minutes and a following cooldown to room temperature for 45 minutes. Unlabeled oligonucleotides were used for crystallizations and ITC. BLI experiments were conducted with a 5′ BiotinTEG-labeled forward oligonucleotide.

### ITC.

ITC measurements were conducted using a MicroCal iTC200 instrument (Malvern Panalytical). Prior to the experiment, DD-DBD protein and unlabeled dsDNA (Table 5) were dialyzed into 20 mM HEPES pH 7.5, 200 mM NaCl, and 1 mM TCEP and centrifuged (16,000*g*, 30 minutes, 4°C). A total of 250 μM dsDNA (RA, P2 WT, P2 -169C>T, P2 -181G>A, P2 -181G>T) was titrated into 50 μM DD-DBD. A program with 19 injections of 2 μL with a 2-minute spacing interval was applied. All measurements were performed at 30°C. Technical replicates were obtained from the same protein batch, on separate measurement days (*n* = 3). Data were analyzed using the MicroCal PEAQ-ITC analysis software (Malvern Panalytical). Titration data were fit to a model assuming 2 sets of binding sites with the ligand in the cell. Δ*G*, Δ*H*, *T*Δ*S, N,* and *K_D_* were extracted from the fits. Statistical analysis was applied to compare *K_D_* values of different variants. *P* values were calculated by performing unpaired 2-tailed *t* tests for each individual pair of oligonucleotides in GraphPad Prism 9.3.0, using the Welch assumption of nonidentical standard deviations.

### BLI.

BLI experiments were performed on an Octet RED96 instrument (FortéBio). Measurements were conducted in dip-and-read mode. All measurements were performed at 30°C in 20 mM HEPES pH 7.5, 200 mM NaCl, 1% BSA, 0.05% Tween 20, and 1 mM TCEP, as technical duplicates. A total of 25 nM of respective BiotinTEG-labeled dsDNA (Table 5) was immobilized on a high-capacity Super Streptavidin Biosensor (Sartorius), followed by a blocking step with 10 μg/mL biocytin (MilliporeSigma). Association and dissociation reactions were simultaneously monitored for 8 analyte concentrations. Purified DD-DBD was applied at ranges 0–3 μM (RA), 0–15 μM (P2 WT), 0–20 μM (P2 -169C>T), or 0–35 μM (P2 -181G>A/T). The association time was 5 minutes (RA) or 2 minutes (P2 WT/variants), while the dissociation time was 10 minutes (RA) or 5 minutes (P2 WT/variants).

Data were analyzed using the Octet Analysis Studio Software Version 9.0 (Sartorius). Curve fits for association and dissociation traces were generated by using the 1:1 homogenous ligand fitting algorithm. *k_obs_* and *k_off_* were extracted from the fits. For a 1:1 binding event and analyte in excess, the following formula (54) describes the relation between the rate constants: *k_obs_* = *k_on_* [DD-DBD] + *k_off_*. Obtained *k_obs_* values were plotted against DD-DBD concentrations, and the data were fitted to a linear function using GraphPad Prism 9.3.0. *k_on_* was extracted from the slope of the linear curve. *k_off_* was obtained by calculating the mean *k_off_* value across the tested concentrations, as *k_off_* is independent of analyte concentration. *k_on_* and *k_off_* were used to calculate the *K_D_* value of the reaction (*K_D_* = *k_off_*/*k_on_*). For the DBD:RA interaction, an additional fitting procedure with a 1:2 heterogenous ligand 3-state binding model was conducted. *k_on,1_*, *k_on,2_*, *k_off,1_*, and *k_off,2_* were computed in the analysis software. An artifact was observed in the dissociation curves for high DD-DBD concentrations with P2 -169C>T, P2 -181G>A, and P2 -181G>T oligonucleotides, which is why these traces were excluded from kinetic analyses.

Additionally, *K_D_* values were calculated from equilibrium responses of the entire [DD-DBD] range. Responses were plotted against DD-DBD concentration and fitted with a single exponential function using GraphPad Prism 9.3.0. The *K_D_* value is defined as the concentration at which 50% of the equilibrium response is reached.

### Crystallization and diffraction data acquisition.

DBD:P2 WT and DBD:P2 -169C>T complexes were crystallized from concentrated ITC samples (10–30 mg/mL) using the sitting-drop vapor-diffusion method at 8°C. DBD:P2 -181G>A and DBD:P2 -181G>T complexes were formed by 2:1 mixing of 300 μM DBD and 150 μM P2 variant oligonucleotides in crystallization buffer (20 mM HEPES pH 7.5, 100 mM NaCl, 1 mM TCEP) and crystallized using the same method. Protein-DNA complexes were mixed with mother liquor (Table 7) on Swissci 96-Well 3-Drop plates (Molecular Dimensions) using a Mosquito LCP (TTP Labtech) nanodispenser. Crystallization drops had a final volume of 300 nL. Crystals were briefly soaked in 25% glycerol for cryoprotection (Table 7), before being flash-frozen in liquid N_2_.

X-ray diffraction data were collected at the EMBL/DESY PETRAIII P13 (55) and the ESRF MASSIF-3 ID30A-3 (56, 57) beamlines at 100°K in remote data collection mode.

### Diffraction data processing and structure refinement.

X-ray diffraction data were processed and scaled with XDS and XSCALE (58), respectively. Molecular replacement, refinement, and structure validation were performed using Phenix (59, 60). Molecular replacement was done with the DBD:RA crystal structure (PDB: 1IC8) (37) using Phaser. The models were refined using phenix.refine (61) and iteratively rebuilt using COOT (62). Structures were validated using MolProbity (63) and deposited in the PDB under the accession codes 8PI8 (P2 WT), 8PI7 (P2 -169C>T), 8PI9 (P2 -181G>A), and 8PIA (P2 -181G>T) (Supplemental Table 3).

Crystal structures were analyzed and visualized using the PyMOL (64), ChimeraX (65, 66), and COOT (62) software.

### Computational predictions.

Computational predictions of variant-mediated changes in transcription factor binding were obtained from the FABIAN-variant prediction server (https://www.genecascade.org/fabian/) (41).

### Transactivation assays.

Transactivation assays were performed by using the Dual-Luciferase Reporter Assay System (Promega). An FL gene in a pGL3 basic vector (Promega) was controlled by an HNF-1A–responsive promoter, such as RA, P2, or variants of P2. pGL3-RA and pGL3-P2 have been described previously (67, 68). P2 variants of interest (-169C>T, P2 -181G>A, -181G>T, -192C>G) were introduced into pGL3-P2 by Q5 site-directed mutagenesis (New England Biolabs), using site-specific primers (Table 8). A pRL-SV40 plasmid (Promega), encoding the constitutively expressed *Renilla* luciferase (RL) gene, was used as internal control. Transcriptional activity of HNF-1A was assessed using a pcDNA3.1-nV5 expression plasmid encoding V5-HNF-1A WT or V5-HNF-1A-P112L (36). A pcDNA™3.1/nV5-DEST vector (Invitrogen) was used as empty vector control.

Transactivation assays were performed in a 24-well format, where cells were seeded at a density of 55,000 cells/well (HeLa) or 250,000 cells/well (MIN6). At 24 hours postseeding, cells were transfected with 6.25 ng pRL-SV40, 0.6 μg pGL3-RA/P2/P2var, and 0.4 μg pcDNA3.1-nV5-WT/V5-P112L/EV, using XtremeGene transfection reagent (Roche), for HeLa, or Lipofectamine 2000 transfection reagent (Invitrogen), for MIN6. At 24 hours following transfection, cells were washed twice with phosphate-buffered saline (pH 7.4) before being lysed and processed according to the assay manual. FL activities were normalized to RL activities, and the obtained values were subsequently normalized to the FL/RL of the samples reporting on respective reference samples.

HeLa cells (CCL-2, ATCC) were grown in DMEM growth medium (MilliporeSigma), supplemented with 15% v/v fetal bovine serum (MilliporeSigma), 4 mM l-glutamine (MilliporeSigma), and 1% penicillin-streptomycin. MIN6 cells (39, 40), provided by Claes Wollheim (Lund University, Lund, Sweden), were cultured in DMEM growth medium (Gibco), supplemented with 15% fetal bovine serum and 1% v/v penicillin-streptomycin (MilliporeSigma). Cells were kept in a humidity incubator at 37°C and 5% atmospheric CO_2_.

### Statistics.

For clinical data analysis, normality of continuous data was tested by QQ-plots and Shapiro-Wilks test. Continuous data are presented as means and standard deviations unless stated otherwise. We constructed Kaplan-Meier curves and used Cox proportional hazard model to analyze differences in age at onset of diabetes. Endpoint was age at onset of diabetes, and patients were censored if they had not developed diabetes at age of investigation. Whether or not the patient had diabetes, age at onset of diabetes or age at investigation as appropriate, sex, and BMI were all included in final multivariate model through forced entry. Statistical significance was considered with *P* < 0.05. Data acquired from transactivation assays are presented as individual data points and means. Statistical testing for differences between the sample groups was performed using a nested 2-tailed *t* test (Figure 3) or a 1-way ANOVA accounting for multiple comparisons (Dunnett T3) (Figure 7), with adjusted *P* < 0.05 considered statistically significant. We used SPSS ver. 29.0 (IBM Corp.) for statistical analyses of clinical data and GraphPad Prism 9.3.0 for the statistical analyses of transactivation assays.

### Study approval.

The Committee for Medical Research Ethics of Western Norway approved the study (2009/2079). Written informed consent was obtained.

### Data availability.

Values for all experimental data points in graphs are reported in the Supporting Data Values file. Raw data on individual mutation carriers are not included because of confidentiality requirements.

## Author contributions

LK, TA, and PRN conceptualized the study. LK performed quality control and conducted laboratory experiments. LK curated and analyzed experimental data. LK, TA, and PK validated experimental results. AR, MM, PK, and LK processed and refined x-ray crystallography data sets. JM, KC, CSM, CA, PD, SP, CVA, and CBC collected and collated clinical data. ET, JM, and PRN analyzed and visualized clinical data. LK visualized experimental data. LK wrote the original draft. All authors reviewed and edited the manuscript. LK, PRN, and TA acquired funding. PK acquired beamtime. LK conducted project management. TA and PRN administered the project. PK, TA, and PRN conducted supervision.

## Supplementary Material

Supplemental data

Supporting data values

## Figures and Tables

**Table 8:**
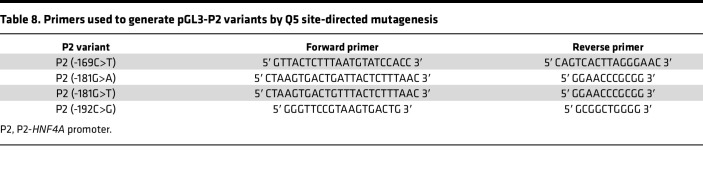
Primers used to generate pGL3-P2 variants by Q5 site-directed mutagenesis

**Table 7:**
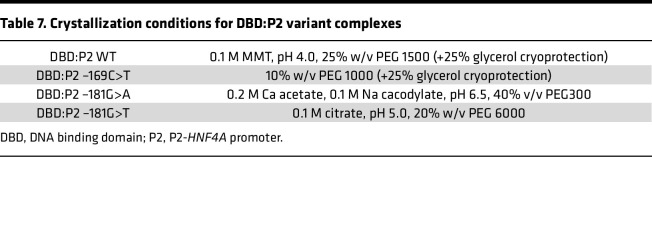
Crystallization conditions for DBD:P2 variant complexes

**Table 1:**
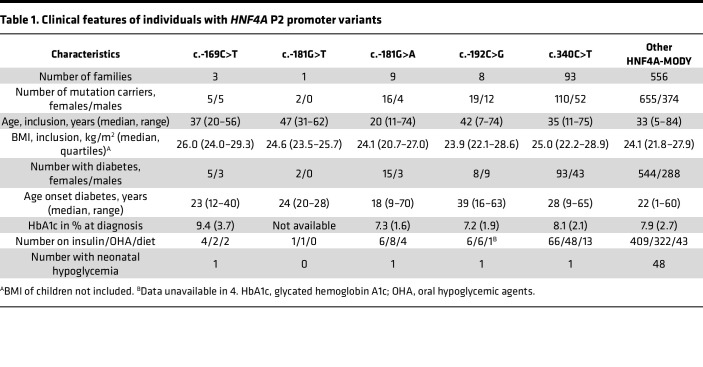
Clinical features of individuals with *HNF4A* P2 promoter variants

**Table 2:**
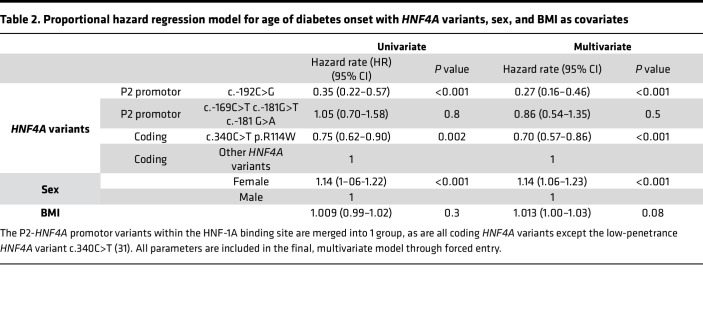
Proportional hazard regression model for age of diabetes onset with *HNF4A* variants, sex, and BMI as covariates

**Table 3:**
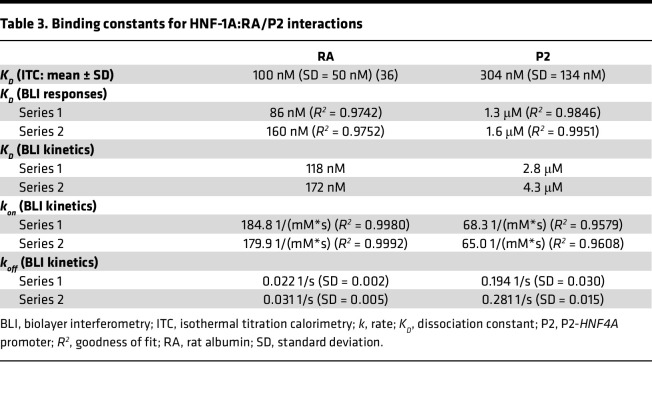
Binding constants for HNF-1A:RA/P2 interactions

**Table 4:**
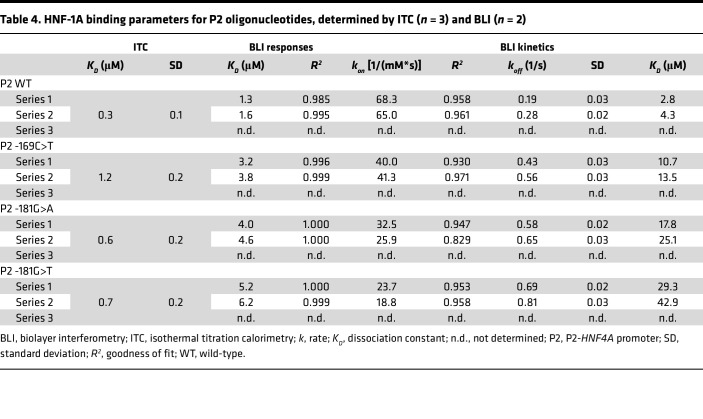
HNF-1A binding parameters for P2 oligonucleotides, determined by ITC (*n* = 3) and BLI (*n* = 2)

**Table 5:**
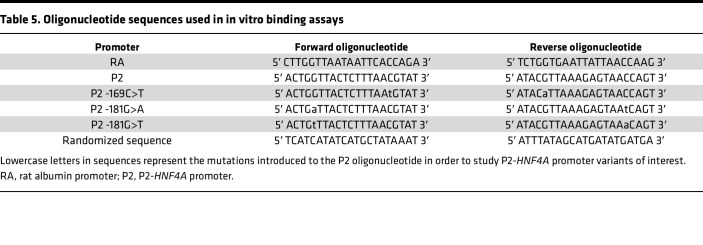
Oligonucleotide sequences used in in vitro binding assays

**Table 6:**
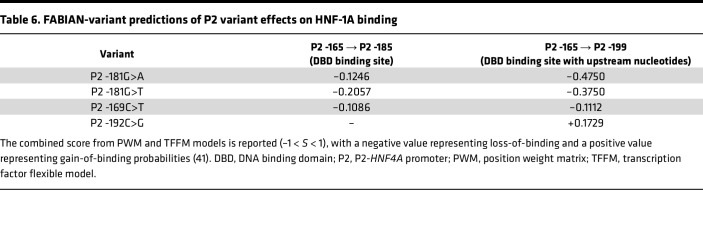
FABIAN-variant predictions of P2 variant effects on HNF-1A binding

## Version 1 06/10/2024

Electronic publication
