# Supplementary material for: Molecular mechanism of HNF-1A–mediated HNF4A gene regulation and promoter-driven HNF4A-MODY diabetes
Source: JCI Insight. 2024 Jun 10;9(11):e175278. doi: 10.1172/jci.insight.175278 (PMC11382887; doi:10.1172/jci.insight.175278)
Supplement: Supplemental data [file jciinsight-9-175278-s041.pdf]

Supplementary information

Molecular mechanism of HNF-1A mediated *HNF4A* gene regulation and promoter-driven HNF4A-MODY diabetes

Laura Kind, Janne Molnes, Erling Tjora, Arne Raasakka, Matti Aukusti Myllykoski, Kevin Colclough, Cécile Saint-Martin, Caroline Adelfalk, Petra Dusatkova, Stepanka Pruhova, Camilla Valtonen-André, Christine Bellanné-Chantelot, Thomas Arnesen, Petri Kursula & Pål Rasmus Njølstad

Fig. S1 Comparison between DBD:RA and DBD:P2 complex formation by BLI.

Fig. S2 Electron density maps for Gln141 in chain A from the DBD:RA (A) and DBD:P2 (B) crystal structures.

Fig. S3. Electron density maps for Lys273 and Asn266 in chain B from the DBD:P2 WT (left) and DBD:P2 -169C>T (right) crystal structures.

Fig. S4. Electron density maps for Lys273 and Asn266 in chain A from the DBD:P2 WT (left), DBD:P2 -181G>A (middle), and DBD:P2 -181G>T (right) crystal structures.

Fig. S5. Pedigrees of 21 families with a P2-*HNF4A* promoter gene variant.

Table S1. Ethnicity of different *HNF4A* mutation carriers.

Table S2. Kinetic parameters of the DD-DBD:RA interaction, extracted from fitting association and dissociation traces to a 1:2 three-state binding model with heterogenous ligand.

Table S3. X-ray diffraction data collection and refinement statistics.

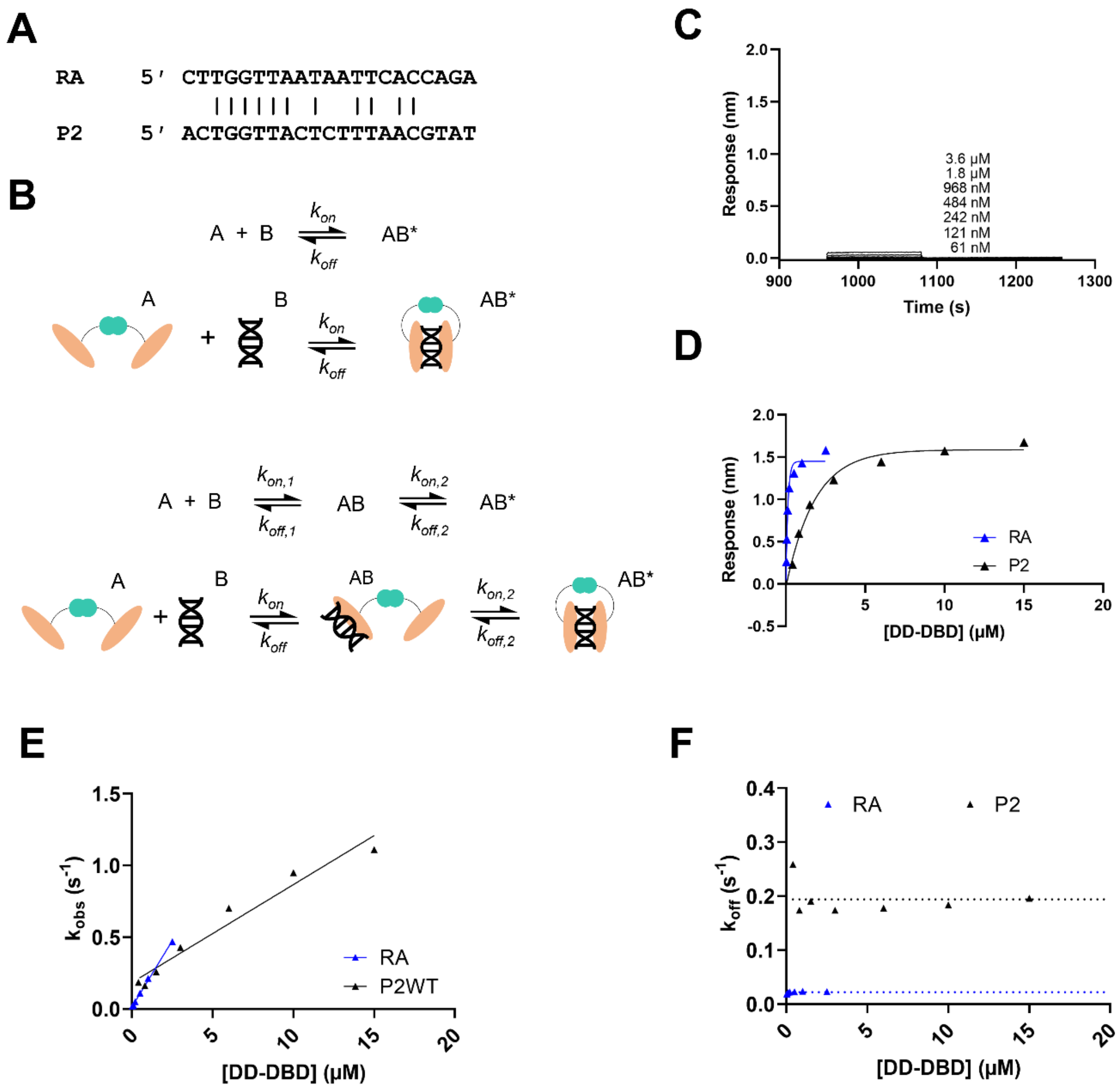

Fig. S1. Comparison between DBD:RA and DBD:P2 complex formation by BLI. A) Sequence alignment between the HNF-1A binding sites in the RA and P2 promoter. Matching nucleotides are indicated by a vertical line. B) Two-state (top) and three-state (bottom) binding reaction models used for fitting of BLI association and dissociation traces. The schematic depicts a suggested association and dissociation mechanism based on established binding interfaces and SAXS solution data of purified DD-DBD (1, 2). In a two-state binding model (top), the two DBD domains of the dimeric DD-DBD protein simultaneously bind to one DNA molecule from opposite sides of the DNA helix. In a potential realization of a three-state binding model, one DBD of the DD-DBD molecule binds to one side of the DNA, followed by binding of the second DBD domain in a cooperative manner (bottom). Considering the high flexibility of the DD-DBD due to disordered linker regions between the DD and DBD domain (2), another scenario could involve one DD-DBD molecule sequentially binding two separate DNA molecules on the surface of the biosensor (not illustrated in this model). C) BLI association and dissociation traces for a control oligo with randomized DNA sequence (Table 6), with the y-axis being in scale with Fig. 2C,D. D-F) BLI data comparing DBD:RA (blue) and DBD:P2 (black) interactions. Measurements were performed as technical duplicates and measurement series 1 is shown representatively. D) BLI response curves for P2 WT (0 – 15 µM DD-DBD), and RA (0 – 2.5 µM) oligonucleotides. E) Observed rate constants (k_{obs}), extracted from association reaction traces in BLI measurements. Data were fitted to a linear function f(x) = m x + n (solid line), for which m corresponds to the association rate (k_{on}) noted in Table 3 (3). F) Dissociation rates (k_{off}), extracted from dissociation reaction traces in BLI measurements. The average value across [DD-DBD] is represented as dotted line and corresponds to k_{off} noted in Table 3 (3).

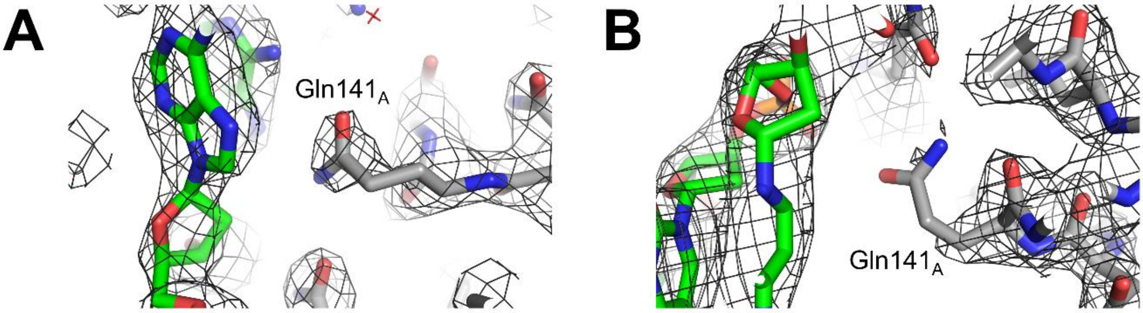

Fig. S2. Electron density maps for Gln141 in chain A from the DBD:RA (A) and DBD:P2 (B) crystal structures. The map contour level was set to σ = 1.5 RMSD for map comparison.

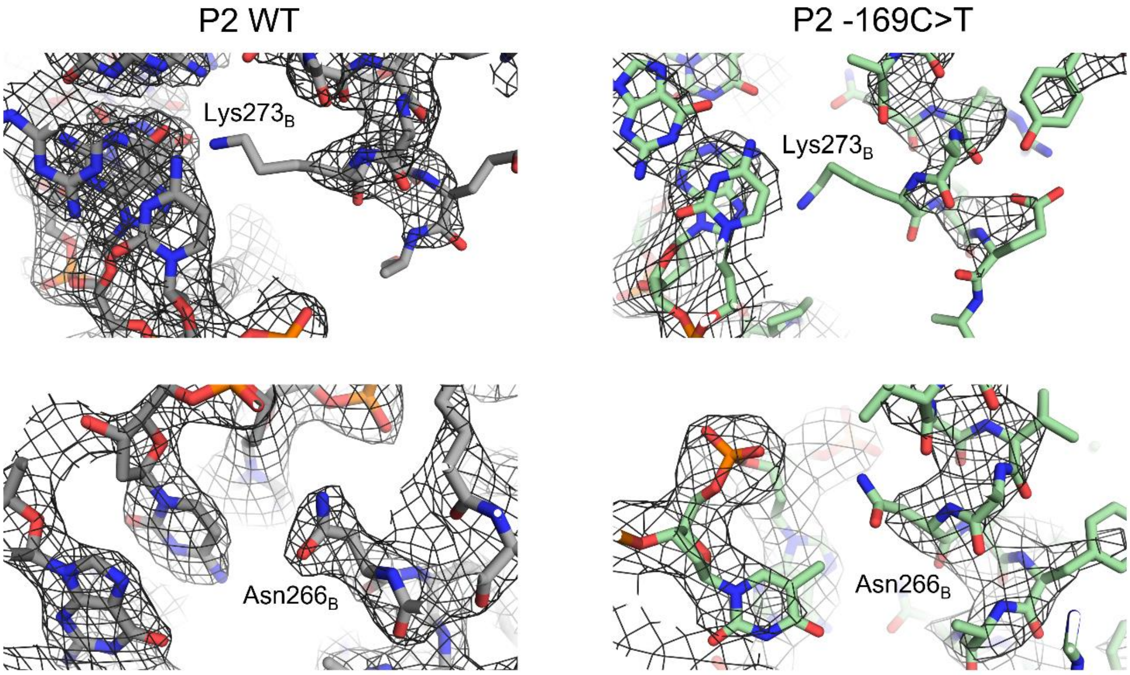

Fig. S3. Electron density maps for Lys273 and Asn266 in chain B from the DBD:P2 WT (left) and DBD:P2 -169C>T (right) crystal structures. The map contour level was set to σ = 1.5 RMSD for map comparison.

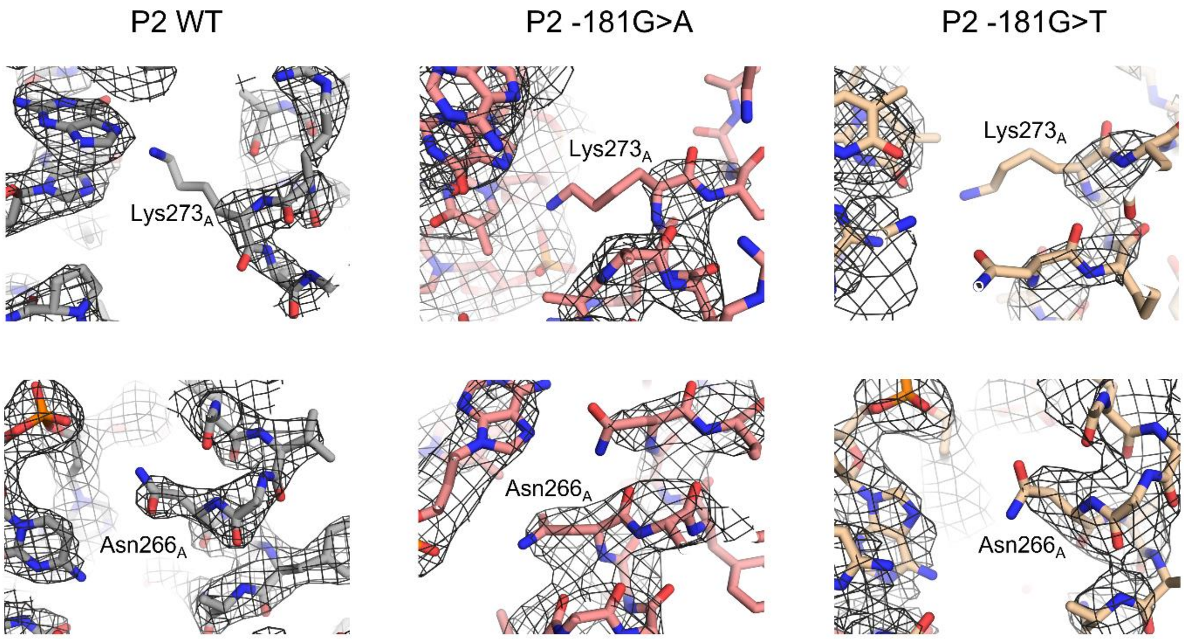

Fig. S4. Electron density maps for Lys273 and Asn266 in chain A from the DBD:P2 WT (left), DBD:P2 -181G>A (middle), and DBD:P2 -181G>T (right) crystal structures. The map contour level was set to σ = 1.5 RMSD for map comparison.

Fig. S5. Pedigrees of 21 families with a P2-*HNF4A* promoter gene variant. Circles represent female family members, squares denote male family members, open symbols indicate unaffected family members, and symbols with a slash denote deceased family members. Solid symbols denote diabetes and grey symbols impaired glucose tolerance. Individual mutation status is given where tested: N, normal allele; M, mutated allele. Arrowheads indicate the probands of each family. Selected pedigrees have been published previously (4-6).

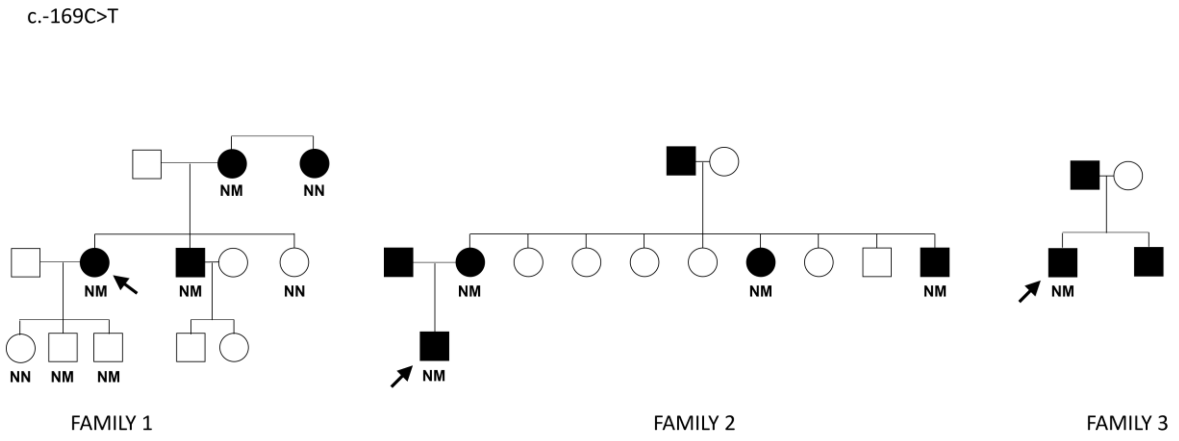

c.-181G>T

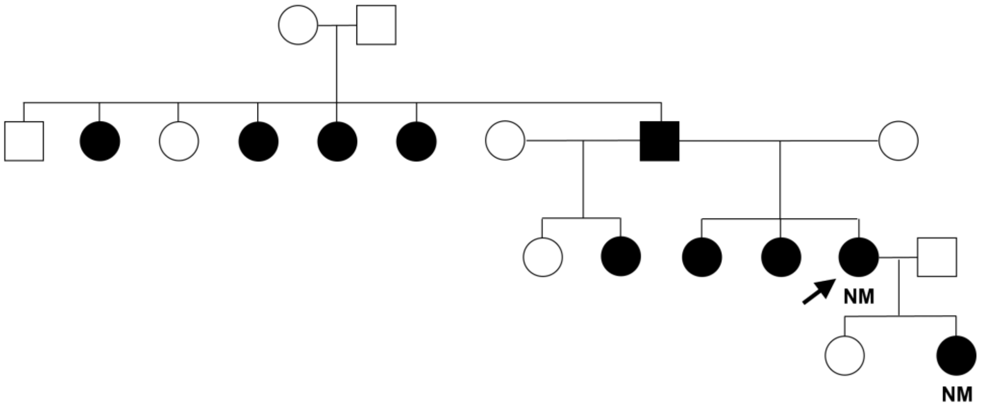

FAMILY 4

c.-181G>A

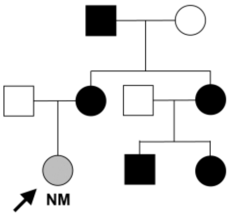

FAMILY 5

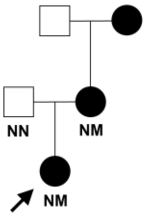

FAMILY 6

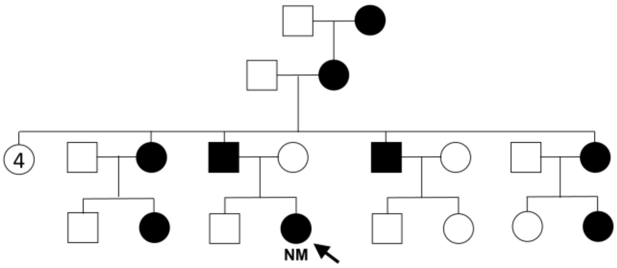

FAMILY 7

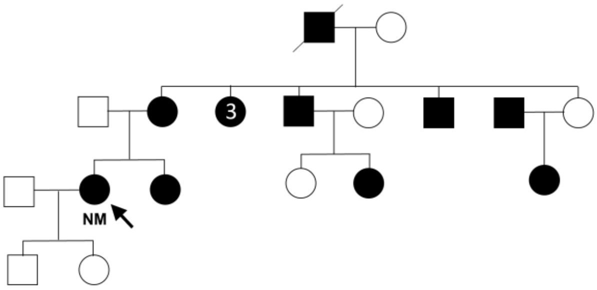

FAMILY 8

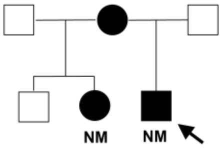

FAMILY 9

c.-181G>A

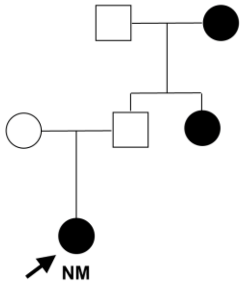

FAMILY 10

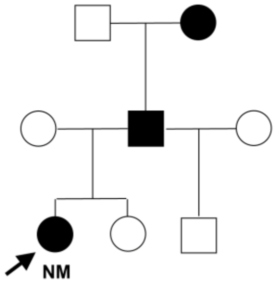

FAMILY 11

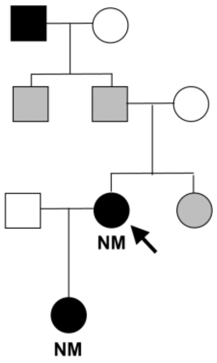

FAMILY 12

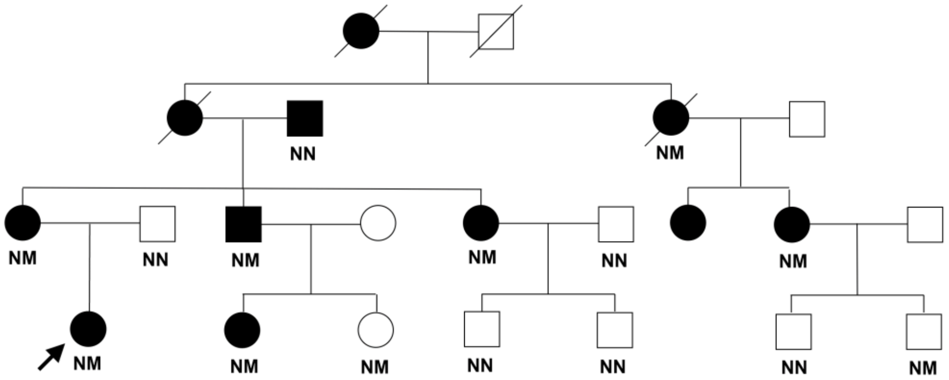

FAMILY 13

c.-192C>G

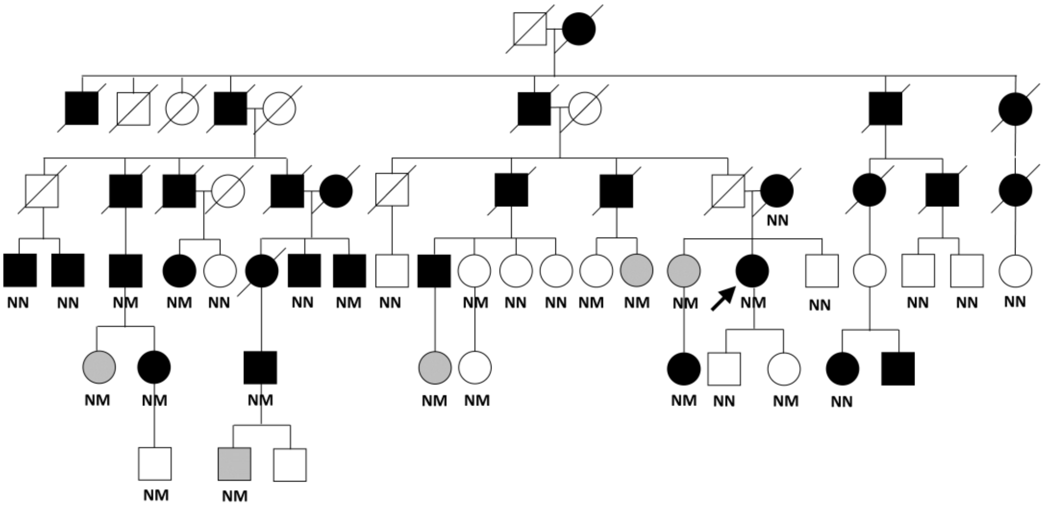

FAMILY 14

c.-192C>G

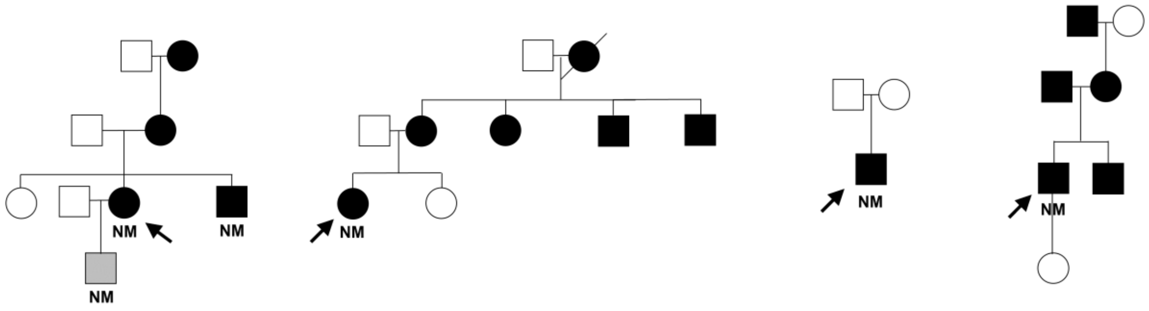

FAMILY 15 FAMILY 16 FAMILY 17 FAMILY 18

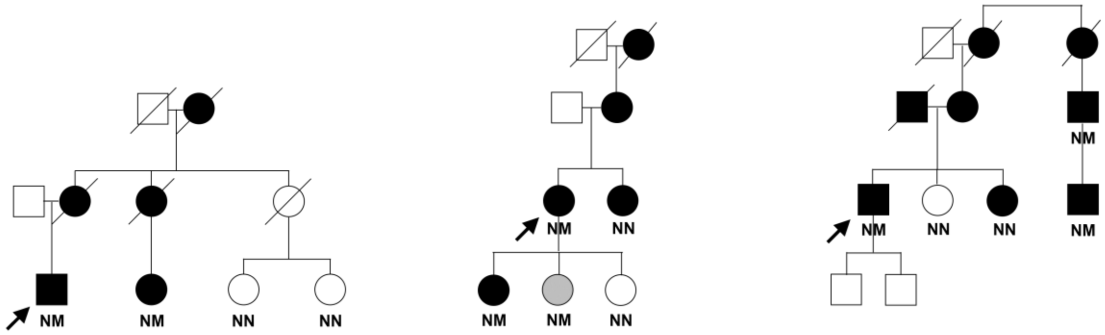

FAMILY 19 FAMILY 20 FAMILY 21

Table S1. Ethnicity of different *HNF4A* mutation carriers.

	**Caucasian**	**African**	**East Asian**	**South Asian**	**Middle East/ North African**	**Other**
**c.-192C>G**	31	0	0	0	0	0
**c.-169C>T, c.-181G>T, c.-181G>A**	21	0	0	0	4	0
**c.340C>T**	144	1	1	0	1	5
**Other *HNF4A* variants**	849	13	43	19	23	17

Table S2. Kinetic parameters of the DD-DBD:RA interaction, extracted from fitting association and dissociation traces to a 1:2 three-state binding model with heterogenous ligand. Results from measurement series 1 are presented representatively. The properties of the dissociation traces suggest that the DD-DBD dissociation from the RA promoter may be of cooperative nature (example model shown in Fig. S1B) but could also stem from artefacts in the measurement. In order to test the molecular basis of this phenomenon, a thorough mutagenesis approach would be required, to systematically test the effect of protein modifications on the association and dissociation rates.

**[DD-DBD] (nM)**	**Response (nm)**	$k_{obs,1}$ **(1/s)**	$k_{obs,2}$ **(1/s)**	$k_{off,1}$ **(1/s)**	$k_{off,2}$ **(1/s)**
25	0.263	1.31E-02	1.31E-02	2.63E-02	2.63E-02
50	0.530	1.42E-02	1.42E-02	2.22E-02	2.22E-02
100	0.875	2.52E-02	2.52E-02	2.16E-02	2.16E-02
200	1.135	5.01E-02	5.01E-02	1.66E-01	2.05E-02
500	1.310	1.08E-01	1.08E-01	1.68E-01	1.96E-02
1000	1.430	2.06E-01	2.06E-01	1.94E-02	1.86E-01
2500	1.581	4.43E-01	4.43E-01	1.91E-01	1.83E-02

Table S3. X-ray diffraction data collection and refinement statistics.

	P2 WT	P2 -169C>T	P2 -181G>A	P2 -181G>T
Beamline	ESRF MASSIF-3 ID30A-3	ESRF MASSIF-3 ID30A-3	EMBL/DESY PETRAIII P13	EMBL/DESY PETRAIII P13
Detector	DECTRIS EIGER X 4M	DECTRIS EIGER X 4M	DECTRIS EIGER X 16M	DECTRIS EIGER X 16M
Wavelength	0.968 Å	0.968 Å	0.976 Å	0.976 Å
Resolution range	48.33 - 2.30 (2.36 - 2.30)	48.14 - 3.20 (3.29 - 3.20)	47.64 - 2.80 (2.92 - 2.80)	47.55 - 2.80 (2.87 - 2.80)
Space group	$P2_12_12_1$	$P2_12_12_1$	$P2_12_12_1$	$P2_12_12_1$
Unit cell (a, b, c, α, β, γ)	46.70, 55.04, 202.07, 90, 90 ,90	49.46, 52.51, 210.28, 90, 90, 90	49.01, 53.07, 202.67, 90, 90, 90	48.69, 48.75, 215.81, 90, 90, 90
Total reflections	177412 (13443)	69688 (5574)	100318 (7482)	110058 (9120)
Unique reflections	24010 (1724)	9655 (729)	13600 (994)	12346 (986)
Multiplicity	7.4 (7.8)	7.2 (7.6)	7.4 (7.5)	8.9 (9.2)
Completeness (%)	99.5 (98.9)	99.6 (99.7)	99.1 (99.6)	92.4 (98.3)
Mean I/σ (I)	6.41 (0.6)	10.86 (1.34)	9.77 (0.43)	7.15 (0.63)
Wilson B-factor	58.67	112.20	101.02	81.36
R-meas (%)	20.3 (345.9)	16.0 (211.3)	11.6 (377.4)	19.9 (429.2)
$CC_{1/2}$ (%)	99.4 (20.6)	99.7 (57.2)	99.7 (30.7)	99.7 (28.9)
Reflections used in refinement	23743 (2164)	9599 (967)	13482 (1229)	12302 (1243)
Reflections used for R-free	1188 (108)	952 (95)	668 (63)	613 (62)
R-work	0.2138 (0.3988)	0.2206 (0.3630)	0.2339 (0.4397)	0.2451 (0.4124)
R-free	0.2607 (0.4114)	0.2804 (0.3881)	0.2934 (0.4475)	0.3000 (0.3943)
Number of non-hydrogen atoms	3651	3607	3657	3545
Number of atoms in macromolecules	3636	3605	3657	3539
Number of ligands	2	2	0	1
Protein residues	338	335	342	326
RMS(bonds)	0.003	0.02	0.004	0.004
RMS(angles)	0.54	0.43	0.71	0.64
Ramachandran favored (%)	98.48	99.39	95.51	98.11
Ramachandran allowed (%)	1.52	0.61	4.19	1.89
Ramachandran outliers (%)	0.00	0.00	0.30	0.00
Rotamer outliers (%)	0.67	0.00	1.34	0.70
Clashscore	6.82	4.57	15.36	14.08
Average B-factor	67.5	132.58	120.12	99.18
Average B-factor macromolecules	68.25	132.0	122.13	96.38
Average B-factor ligands	69.45	121.15		84.24
PDB ID	8PI8	8PI7	8PI9	8PIA

References – Supplementary information

1. Chi YI, Frantz JD, Oh BC, Hansen L, Dhe-Paganon S, Shoelson SE. Diabetes mutations delineate an atypical POU domain in HNF-1alpha. Mol Cell. 2002;10(5):1129-37.
2. Kind L, Raasakka A, Molnes J, Aukrust I, Bjorkhaug L, Njolstad PR, et al. Structural and biophysical characterization of transcription factor HNF-1A as a tool to study MODY3 diabetes variants. J Biol Chem. 2022;298(4):101803.
3. Karlsson E, Jemth P. Kinetic Methods of Deducing Binding Mechanisms Involving Intrinsically Disordered Proteins. Methods Mol Biol. 2021;2263:105-33.
4. Wirsing A, Johnstone KA, Harries LW, Ellard S, Ryffel GU, Stanik J, et al. Novel monogenic diabetes mutations in the P2 promoter of the HNF4A gene are associated with impaired function in vitro. Diabet Med. 2010;27(6):631-5.
5. Raeder H, Bjorkhaug L, Johansson S, Mangseth K, Sagen JV, Hunting A, et al. A hepatocyte nuclear factor-4 alpha gene (HNF4A) P2 promoter haplotype linked with late-onset diabetes: studies of HNF4A variants in the Norwegian MODY registry. Diabetes. 2006;55(6):1899-903.
6. Hansen SK, Parrizas M, Jensen ML, Pruhova S, Ek J, Boj SF, et al. Genetic evidence that HNF-1alpha-dependent transcriptional control of HNF-4alpha is essential for human pancreatic beta cell function. J Clin Invest. 2002;110(6):827-33.
